# Molecular Interface of S100A8 with Cytochrome *b_558_* and NADPH Oxidase Activation

**DOI:** 10.1371/journal.pone.0040277

**Published:** 2012-07-10

**Authors:** Sylvie Berthier, Minh Vu Chuong Nguyen, Athan Baillet, Marc-André Hograindleur, Marie-Hélène Paclet, Benoît Polack, Françoise Morel

**Affiliations:** 1 Groupe de Recherche et d’Etude du Processus Inflammatoire (GREPI), Laboratoire “Aging Imaging Modeling” (AGIM), Formation de Recherche en évolution (FRE) Centre National de la Recherche Scientifique CNRS 3405, Université Joseph Fourier UJF, Grenoble, France; 2 Clinic of Rheumatology, Centre Hospitalier Universitaire (CHU), Grenoble, France; 3 « Laboratoire des Enzymes et des Protéines », Centre Hospitalier Universitaire (CHU), Grenoble, France; 4 « Institut de Biologie et Pathologie », Centre Hospitalier Universitaire (CHU), Grenoble, France; 5 Techniques de l’Ingénierie Médicale et de la Complexité–Informatique, Mathématiques et Applications de Grenoble (TIMC-IMAG) Unité Mixte de Recherche (UMR) 5525 Centre National de la Recherche Scientifique (CNRS), Université Joseph Fourier UJF, Grenoble, France; Griffith University, Australia

## Abstract

S100A8 and S100A9 are two calcium binding Myeloid Related Proteins, and important mediators of inflammatory diseases. They were recently introduced as partners for phagocyte NADPH oxidase regulation. However, the precise mechanism of their interaction remains elusive. We had for aim (i) to evaluate the impact of S100 proteins on NADPH oxidase activity; (ii) to characterize molecular interaction of either S100A8, S100A9, or S100A8/S100A9 heterocomplex with cytochrome *b*_558_; and (iii) to determine the S100A8 consensus site involved in cytochrome *b*_558_/S100 interface. Recombinant full length or S100A9-A8 truncated chimera proteins and ExoS-S100 fusion proteins were expressed in *E. coli* and in *P. aeruginosa* respectivel*y*. Our results showed that S100A8 is the functional partner for NADPH oxidase activation contrary to S100A9, however, the loading with calcium and a combination with phosphorylated S100A9 are essential *in vivo*. Endogenous S100A9 and S100A8 colocalize in differentiated and PMA stimulated PLB985 cells, with Nox2/gp91^phox^ and p22^phox^. Recombinant S100A8, loaded with calcium and fused with the first 129 or 54 N-terminal amino acid residues of the *P. aeruginosa* ExoS toxin, induced a similar oxidase activation *in vitro,* to the one observed with S100A8 in the presence of S100A9 *in vivo*. This suggests that S100A8 is the essential component of the S100A9/S100A8 heterocomplex for oxidase activation. In this context, recombinant full-length rS100A9-A8 and rS100A9-A8 truncated 90 chimera proteins as opposed to rS100A9-A8 truncated 86 and rS100A9-A8 truncated 57 chimeras, activate the NADPH oxidase function of purified cytochrome *b*_558_ suggesting that the C-terminal region of S100A8 is directly involved in the molecular interface with the hemoprotein. The data point to four strategic ^87^HEES^90^ amino acid residues of the S100A8 C-terminal sequence that are involved directly in the molecular interaction with cytochrome *b_558_* and then in the phagocyte NADPH oxidase activation.

## Introduction

Myeloid-Related Proteins (MRP), S100A8 (MRP8) and S100A9 (MRP14), are two calcium-binding proteins of the multigenic S100 family, specifically linked to innate immunity [1]. They are mainly expressed in cells of myeloid origin such as neutrophils or monocytes, but are absent in Epstein-Barr-Virus (EBV) immortalized B lymphocytes [2]. They were also identified in epithelial cells and keratinocytes [3], [4]. In phagocytes, S100A8 and S100A9 associate to form physiologically oligomeric structures (dimer or tetramer) that bind polyunsaturated fatty acids such as arachidonic acid in a calcium dependent manner [5]. The *in vivo* S100A8/S100A9 heterocomplex, named S100A8/S100A9, accounts for the entire arachidonic acid-binding capacity of neutrophil cytosol. The fatty acid carboxyl group is bound by consecutive histidine residues within the unique C-tail of S100A9 [6].

S100 proteins such as S100A8 and S100A9, form non-covalent and antiparallel associated S100A8/S100A9 complexes *in vivo*, which exhibit various functional intra and extra cellular properties [7]. In neutrophils, they are involved in cell differentiation, inhibition of casein kinase II, and trans-endothelial migration. But one of their main functions is to be partners of phagocyte NADPH oxidase regulation [8]. Indeed, S100A8/S100A9 favors the activation of NADPH oxidase. The process is dependent on the transfer of arachidonic acid to plasma membrane level onto cytochrome *b*_558_; during this transfer, S100A8/S100A9 associates with p67^phox^ and Rac [9], [10]. p67^phox^ translocates with other associated cytosolic factors, p47^phox^ and p40^phox^, at the plasma membrane to form an active NADPH oxidase complex with cytochrome *b_558_*. Moreover, the relevant role of S100A8 and S100A9 in the oxidative response depends on the presence of calcium and on a phosphorylation-dependent translocation to plasma membrane where NADPH oxidase assembles and is activated [11], [12], [13]. S100A8 and S100A9 are also secreted outside of the cells through a Golgi-independent pathway [14]. They bind to heparin sulfate proteoglycans resulting in accumulation at the endothelium surface of extracellular complexes of S100A8 and S100A9 that interact with specific binding sites. In this context, both proteins were reported at local sites of inflammation, [15], [16] in neoplastic tumors [17], and in skin diseases [3], where they trigger a crucial danger signal. The term “Alarmin” was introduced recently for these molecules [18]. However S100A8, and to some extent S100A9, are also particularly susceptible to oxidation: recent data suggest that post-translational oxidative modifications of both S100 proteins may act as a regulatory switch and have an important protective role in inflammation [19]. An oligomeric structure of S100A8/S100A9 was reported: while S100A8/S100A9 heterodimers are formed in the absence of calcium, a tetramer structure construction is strictly calcium dependent [20], and was recently crystallized [21]. S100A8 and S100A9 contain two calcium-binding sites of EF-hand type; the heterodimer binds four Ca^2+^ ions. Besides calcium, S100A8 and S100A9 are also able to bind zinc, but the binding requires a heterodimer structure that is necessary to form a zinc-binding site. The heterotetramer displays a high affinity for Zn^2+^: two HXXXH motifs per dimer (residues 83–87) in S100A8 and (residues 91–95) in S100A9 were suggested to be responsible for Zn^2+^ binding. The HXXXH motifs are located at the interface between the respective S100 sub-units; a similar motif was also involved for the zinc-dependent matrix metalloproteinase activity [1]. However, it was reported that the binding of zinc could reverse the calcium-induced arachidonic acid-binding capacity of S100A8/S100A9 heterocomplex [5]. Two prominent roles were assigned to S100A8/S100A9 hetero-oligomers : not only a role in regulation of inflammatory processes, immune response, and wound repair, but also a role in zinc sequestration, inhibiting zinc-dependent enzymes, as well as microbial growth [21].

Nox2 is the prototype of the NADPH oxidase Nox-Duox family, from which 7 members were characterized in humans [22]. In neutrophils, cytochrome *b_558_* is the redox core of NADPH oxidase [23], [24] and the membrane anchorage site for assembly with cytosolic factors, p67^phox^, p47^phox^, p40^phox^, and Rac1/2. NADPH oxidase is unassembled and inactive in resting cells, but upon stimulation by inflammatory mediators or during phagocytosis, the phosphorylation of phox proteins induces intra and intermolecular rearrangements that stabilize all the partners as an oxidase complex at the plasma membrane. This gives an optimal cytochrome *b*_558_ conformation and NADPH oxidase activity.

An allosteric regulation of NADPH oxidase activity was reported [25]: gp91^phox^ or Nox2, was introduced as the catalytic sub-unit of cytochrome *b_558_*, while p22^phox^ may stabilize the Nox2/p22^phox^ heterodimer. The cytosolic activating factors are regulatory effectors: p47^phox^ behaving as an adaptor, while the interaction between p67^phox^ and Nox2 initiates both assembly and activation. In fact, the binding of p67^phox^ to cytochrome *b*_558_, mediates the transition from an inactive to an active conformation of the hemoprotein [26]. Recent data suggested that S100A8/S100A9 could be new allosteric effectors of NADPH oxidase activation in neutrophils [2]; they might potentiate activation of NADPH oxidase and radical oxygen species production. However the mechanisms by which the S100A8/S100A9 heterocomplex increases NADPH oxidase activity remain unknown.

The present article emphasizes the respective functions of S100A8 and S100A9 in neutrophils, and their role in NADPH oxidase activation. Our data confirm that S100A8 is the privileged interactive partner of cytochrome *b*_558._ Furthermore, we show that four amino-acid residues of the C-terminal sequence of S100A8 are directly involved in NADPH oxidase activation. But the *in vivo* interaction of S100A8 with cytochrome *b*_558_ proceeds through a strategic calcium mediated 3D structure conformation involving both S100A8 and S100A9.

## Results

### Generation of Monoclonal Antibodies Against S100 Proteins

**Figure 1:**
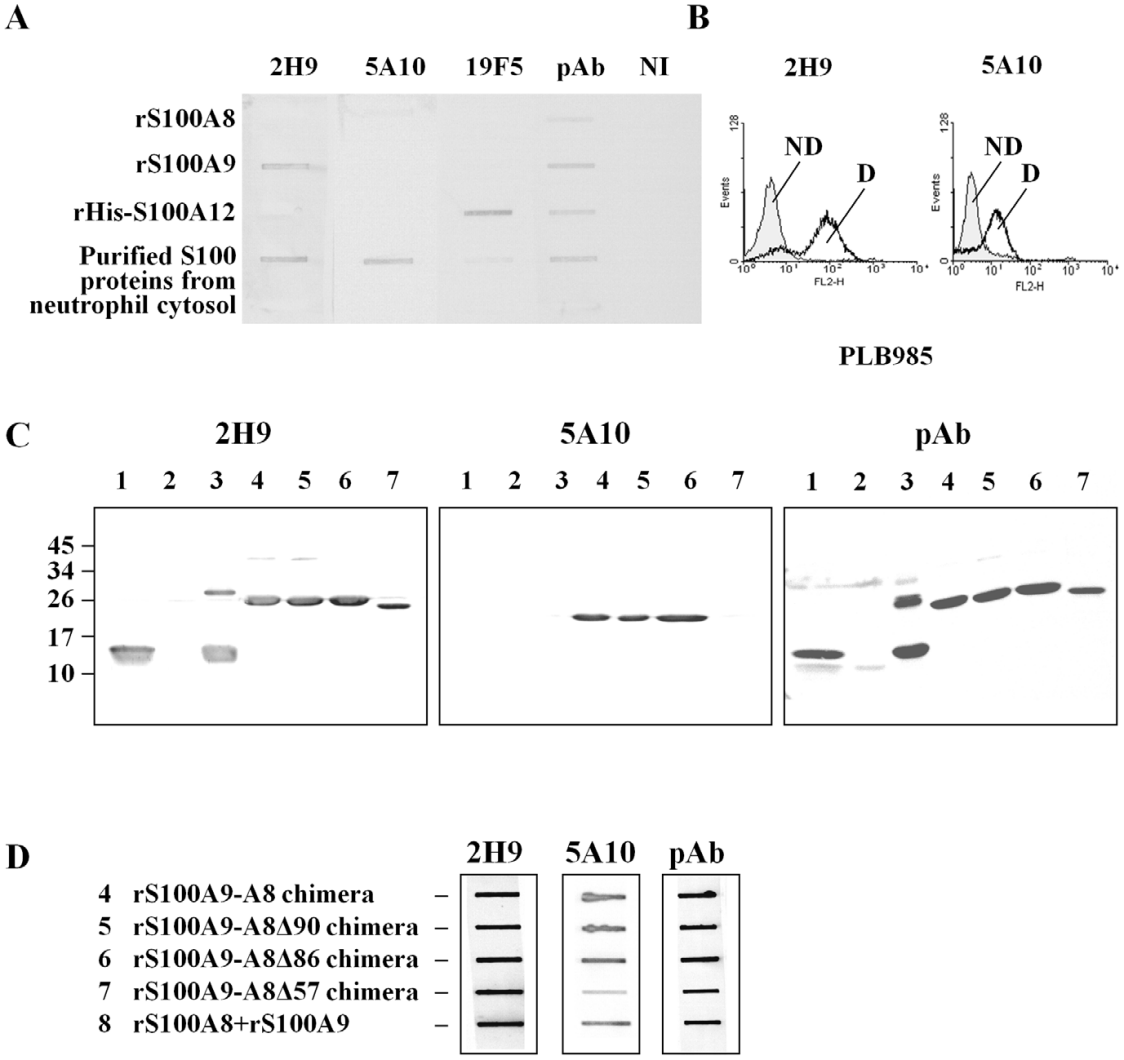
Characterization of two new monoclonal antibodies raised against purified S100 proteins from cytosol of human neutrophils: Validation of recombinant chimera proteins. Monoclonal antibodies were purified from ascetic fluid after mice immunization as described in Materials and Methods. Two monoclonal antibodies (2H9 and 5A10) were characterized by Slot blot (A) against 1.25 ug of rS100A8, rS100A9 and rHis-S100A12, expressed in *E. coli,* S100 proteins purified from neutrophil cytosol [2]. Recombinant His-S100A12 (Figure S1) was used as control and specifically labeled by a commercial monoclonal 19F5 antibody. The results with 5A10 and 2H9 were compared to the ones obtained with rabbit polyclonal antibodies purified from neutrophil cytosol (pAb). Specificity of the mAbs was assessed by FACS (B) in PLB985 cells differentiated (D) or not (ND). Monoclonal antibodies 5A10 and 2H9 were used to validate recombinant chimera proteins by Western blot (C) and Slot blot (D) on neutrophil cytosol (1), recombinant r100A8 (2), rS100A9 (3), rS100A9-A8 chimera (4), rS100A9-A8Δ90 chimera (5), rS100A9-A8Δ86 chimera (6), rS100A9-A8Δ57 chimera (7) and rS100A8 plus rS100A9 (8). 5 ug of protein were loaded in (D). Immune complexes were detected by ECL as described in Materials and Methods section.

In order to study the function of S100 proteins, we first generated monoclonal antibodies raised against S100A8 and S100A9 by intra-peritoneal injection of 4 mice with S100 proteins purified from cytosol of human neutrophils. The IgGs were produced from hybridoma in ascetic fluid and were purified as described in Material and Methods. The specificity of two selected clones, 5A10 (IgG1) and 2H9 (IgG2a), was controlled referring to rS100A8, rS100A9, or S100 proteins purified from cytosol of neutrophils, and rS100A9-A8 chimera proteins. Moreover, a purified rHIS-S100A12 expressed in *E.coli* was used as negative control (Figure S1A) and identified by Western blot with monoclonal antibodies anti-S100A12 (19F5), and anti-histidine (Figure S1B and Figure 1A). 2H9 and 5A10 antibodies both recognized native S100 proteins in the neutrophil cytosol and in differenciated PLB985 cells (Figure 1A, B) but not rS100A12 proteins. Additionally, 2H9 antibody labeled specifically native (Figure 1A) or denatured (Figure 1C, lane 3) rS100A9 but not rS100A8. On the contrary, 5A10 bound only native S100 proteins prepared from cytosol of neutrophils but not rS100A8 or rS100A9 (Figure 1A). Furthermore, 5A10 antibody seemed to recognize S100 proteins only when they were in their native (Figure 1A, in the neutrophil cytosol and Figure 1C, lane 8) or chimera (Figure 1C, line 4) dimerisation states but not when S100 proteins are in monomer status. These results suggest that 5A10 is a conformational antibody and therefore that rS100A9-A8 chimera protein may be in a correct native 3D-like conformation. Finally, as shown on lane 7 of the figure 1C and on lane 7 of the figure 1D, the rS100A9-A8Δ57 chimera protein was not labeled by 5A10 which suggests that the epitope targeted by this antibody could be located between the 86 and 57 amino acid residues of S100A8. A polyclonal antibody (pAb), recognizing both S100A8 and S100A9, was used as a control.

### *Ex vivo* S100 Protein Delivery by *P. aeruginosa* Type Three Secretion System (TTSS) into EBV-B Lymphocyte Cells Stimulates NADPH Oxidase Acticity

In a previous work, we have shown that the NADPH oxidase activity was enhanced after transfection in EBV-B lymphocytes of the genes encoding for S100A8 and S100A9 [2]. To further confirm this finding and to investigate the impact of S100A8 and S100A9 alone or as heterodimer on NADPH oxidase activity, we decided to use the TTSS of Gram (-) *P. aeruginosa* to deliver both proteins inside the cytosol of EBV-B lymphocytes. This delivery system was previously successfully used to reconstitute a functional NADPH oxidase in p67^phox^ deficient EBV-B lymphocytes of Chronic Granulomatous Disease patients by injecting ExoS129-p67^phox^ [27]. Furthermore, the EBV-B lymphocytes constitute an appropriate cellular model for this study since they are totally devoid of S100A8 and S100A9, and also because they display a very low NADPH oxidase activity [2].

**Figure 2:**
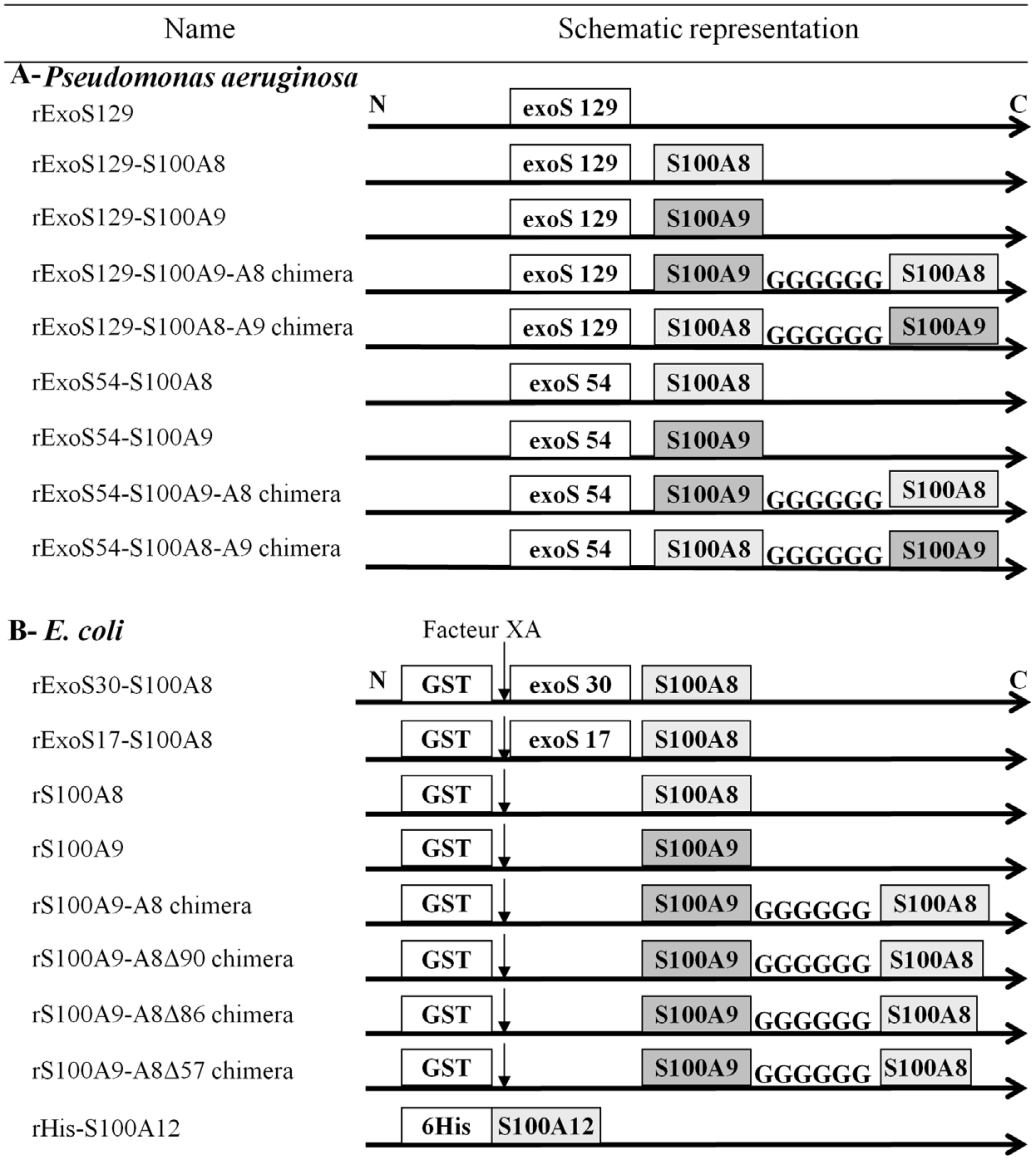
Schematic representation of the recombinant proteins. (A) Proteins expressed in *Pseudomonas aeruginosa*. The name of recombinant proteins matched with the represented schematic constructions. S100A8 and S100A9 are in frame with ExoS129 or ExoS54. Chimera proteins are the result of the binding of S100A8 with S100A9 by 6 Glycine amino acid residues. (B) In E. coli, recombinant S100 proteins are fused to GST and produced by a pGEX5x2 plasmid. The GST cleavage is performed by the XA factor. Some S100A9-A8 chimera proteins are truncated (Δ) at the C-terminal part of S100A9-A8, at amino acid 90 (Δ90), 86 (Δ86) or 57 (Δ57) compared to the full length S100A9-A8.

**Figure 3:**
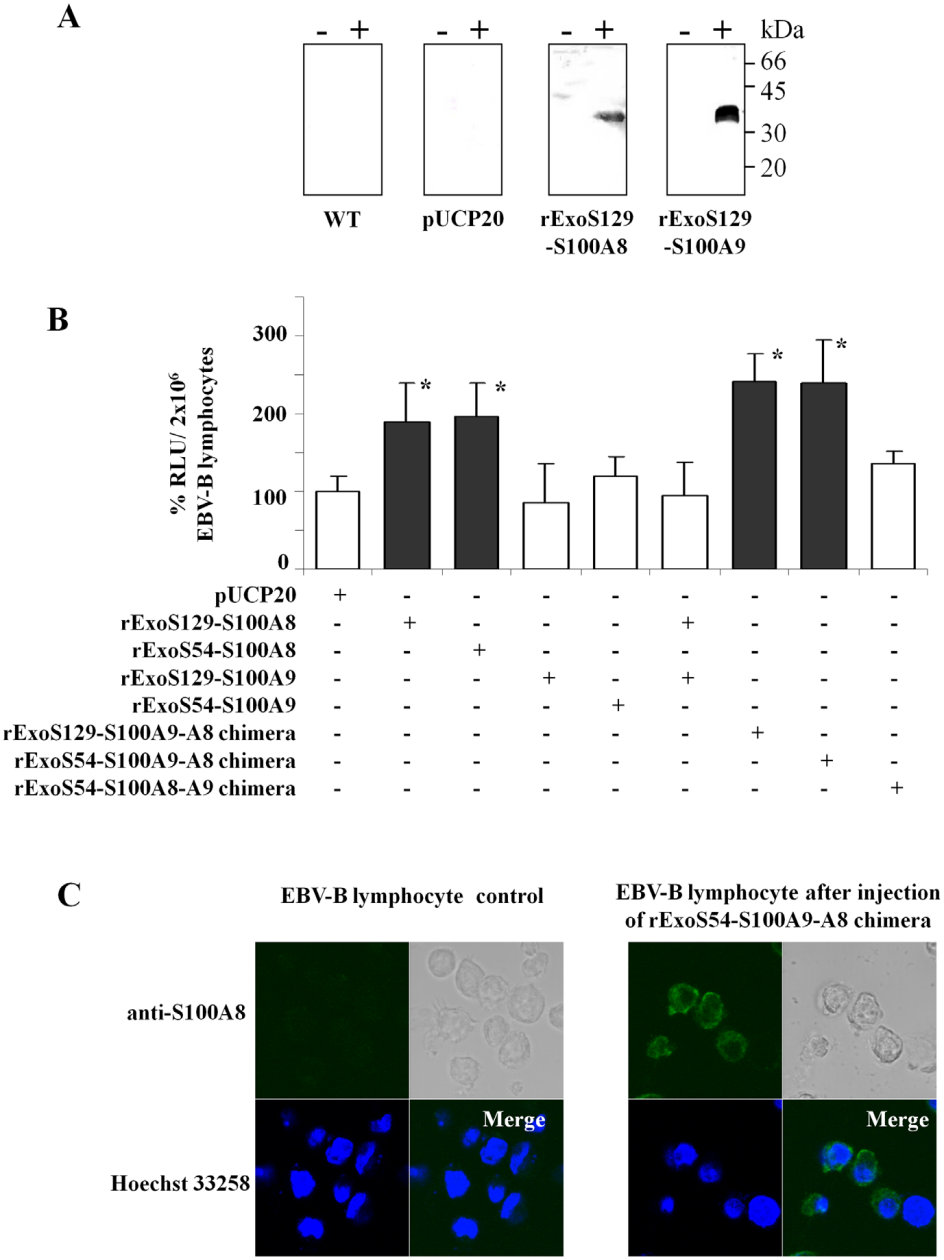
Delivery of ExoS-S100 proteins by Pseudomonas aeruginosa Type Three Secretion System (TTSS) increased NADPH oxidase activity of EBV-B lymphocytes. (A) The *P. aeruginosa* wild type strain or the one transformed with empty plasmid pUCP20 or pUCP20 containing cDNA encoding for rExoS129-S100A8 or rExoS129-S100A9 were induced *in vitro* upon calcium depletion by 5 mM EGTA as described in Materials and Methods. Recombinant ExoS129-S100A8 and rExoS129-S100A9 secreted by TTSS in the culture medium, were detected by Western blot with rabbit polyclonal antibodies raised against purified S100 proteins of neutrophils cytosol as described previously [25] and in Materials and Methods. Immune complexes were stained by ECL. (B) TTSS properties of *P. aeruginosa* were induced *ex vivo* by the contact between CHA strains and EBV-B lymphocytes (MOI 10) in the presence of 10% (v/v) human AB serum (RPMI 1640 medium,) in order to deliver recombinant ExoS-S100 fusion proteins (rExoS129-S100A8, rExoS129-S100A9, rExoS54-S100A8, rExoS54-S100A9, ExoS129-S100A9-A8, ExoS54-S100A9-A8, or ExoS54-S100A8-A9 chimeras). In some experiments, bacteria CHA S100A9 and CHA S100A8 were concomitantly induced by contact for 90 min at 37°C with EBV-B lymphocytes (MOI 5 each) in RPMI 1640 medium. NADPH oxidase activity of 2×10^6^ EBV-B lymphocytes, collected after injection of fusion proteins by TTSS, was measured by chemiluminescence, upon stimulation by 130 nM PMA. Activity was expressed as RLU. Number of experiments n = 3 to 5. Data represent means±SD. *p<0.05, significant difference versus control (NADPH oxidase activity of EBV-B lymphocytes after contact with pUCP20 transfected *P. aeruginosa*). (C) EBV-B lymphocytes, either control cells or cells after rExoS 54-S100A9-A8 chimera injected by *P. aeruginosa,* were fixed, permeabilized, and labeled with goat polyclonal antibody anti human S100A8, C19 (dilution 1∶1,000) for confocal microscopy analysis, as described in Materials and Methods. An Alexa Fluor 488 anti-goat antibody was used to detect the fusion proteins, stained in green, in cells when rExoS54-S100A9-A8 proteins were present. EBV-B lymphocyte nuclei were stained in blue with Hoechst 33258.

rExoS-S100A8 and rExoS-S100A9 fusion proteins were constructed in frame with the first 54 or the first 129 N-terminal amino acid residues of Exotoxin S (ExoS) toxin sequence, from *P. aeruginosa,* as described in the Figure 2. An adequate folding status was maintained by a specific interaction with chaperone Orf-1 (design of constructions in Table S1) [28] and [29]. We first verified that rExoS-S100A8 and rExoS-S100A9 were effectively secreted by the *P. aeruginosa* CHA strain. After induction by calcium-depletion, the fusion proteins with an apparent molecular mass of ∼30–35 kDa were identified by specific polyclonal antibodies raised against S100 proteins of neutrophil cytosol and were found only in the extracellular medium of induced *P. aeruginosa* (Figure 3A). We next used *P. aeruginosa* CHA-S100A8 and CHA-S100A9 to deliver the hybrid fusion rExoS129 or rExoS54 proteins into the cytosol of normal EBV-B lymphocytes. NADPH oxidase activity of (phorbol-myristate-acetate) PMA-stimulated EBV-B lymphocytes was then measured by chemiluminescence. There was no enhancement of oxidase activity of EBV-B lymphocytes over the control value when CHA strain was transformed with the empty vector pUCP20 or with CHA-ExsA-, a mutated strain unable to secrete proteins (not shown). Interestingly, we observed an increase of the NADPH oxidase activity after the delivery of rExoS129-S100A8 into EBV-B lymphocytes (Figure 3B) but not for rExoS-S100A9. It can be also observed that injection of both rExoS-S100A9 and rExoS-S100A8 had no positive activation of oxidase mediated by rExoS-S100A8 on its own, probably because of an unbalanced secreted rExoS-S100A9/rExoS-S100A8 ratio. Indeed, the amount of rS100A9 secreted by *P. aeruginosa* CHA strain (Figure 3A) or expressed in *E.coli* (data not shown) is higher than for rS100A8. However, a negative effect of rExoS129-S100A9 proteins on the conformation of rExoS-S100A8 could not be excluded.

In order to keep a 1/1 S100A9/S100A8 ratio, we decided to generate fusion S100A8 and S100A9 chimera proteins in frame with the ExoS54 or ExoS129: one with the S100A9 on the N terminal side (rS100A9-A8) and one on the C terminal side (rS100A8-A9) (Figure 2). A significant activation of NADPH oxidase was obtained with rExoS129-S100A9-A8 chimera delivery contrary to rExoS129-S100A8-A9 showing a preferential orientation of the chimera with S100A8 at the C terminal side in order to have a correct functional conformation (Figure 3B). Those results were confirmed with ExoS54-S100 fusion proteins: both rExoS54-S100A8 and rExoS54-S100A9-A8 chimera but not the rExoS54-S100A8-A9 increased NADPH oxidase activity of EBV-B lymphocytes after delivery of fusion proteins (Figure 3B). The presence of rExoS54-S100A9-A8 chimera after delivery by *P. aeruginosa* to EBV-B lymphocytes was controlled by confocal microscopy (Figure 3C). The injected rExoS54-S100A9-A8 chimera protein was partitioned between cytosol and plasma membrane of the EBV-B cells. 129 ExoS toxin of CHA strain toxicity was addressed as shown in Table S2 and the results indicate that there was no toxic effect of the toxin before 2.5 h of contact.

### Effects of ExoS-S100 Fusion Proteins on NADPH Oxidase Turnover Measured in a Cell-free Assay with Purified Cytochrome *b_558_*

**Figure 4:**
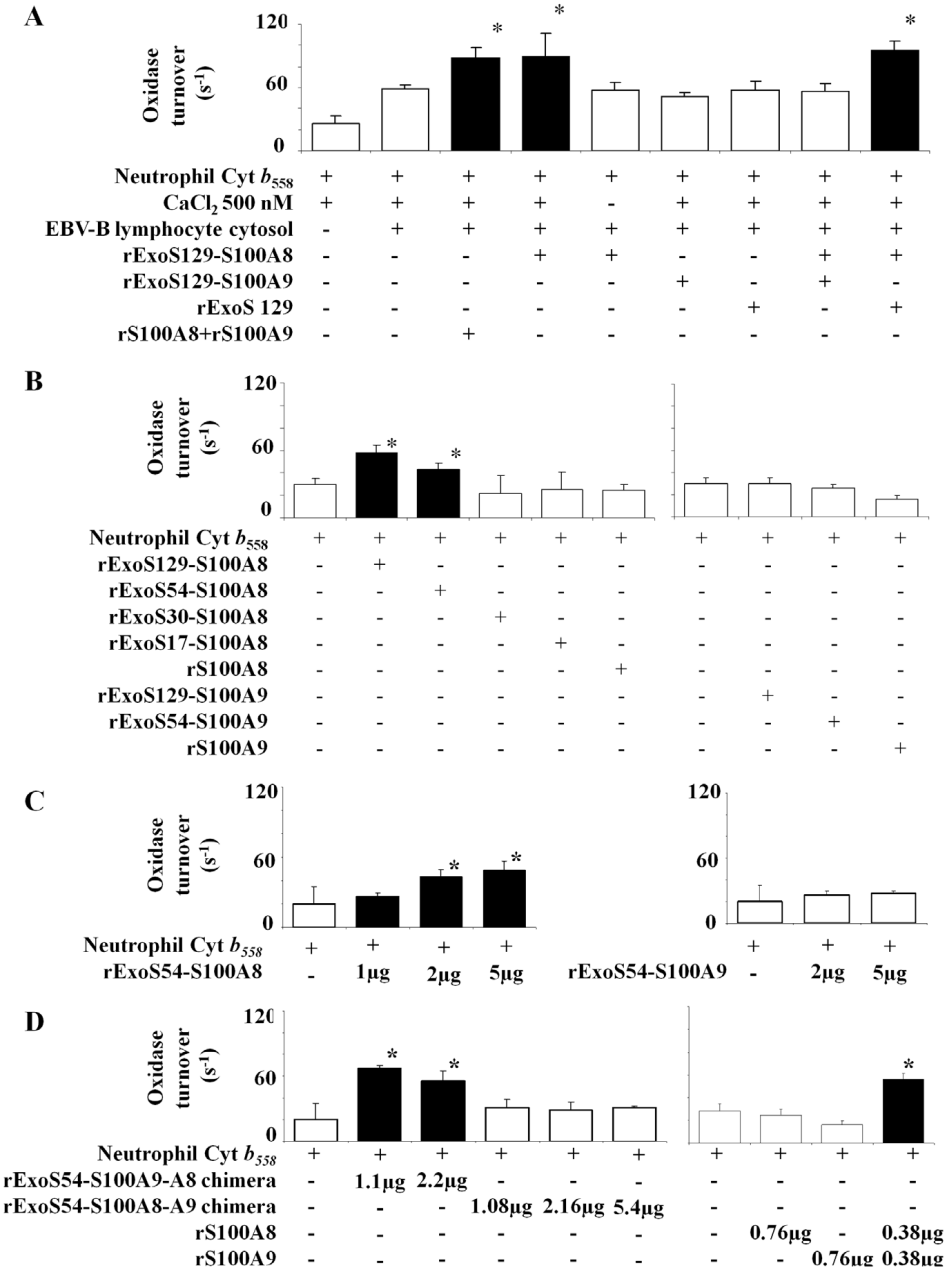
Effect of recombinant S100A8 and rS100A9 expressed as ExoS fusion proteins on NADPH oxidase activity measured in vitro in a cell-free assay. (A) Recombinant rExoS129-S100 fusion proteins, rS100A8 and rS100A9 were used in a cell free assay as follows with the purified cytochrome *b*_558_ from human neutrophils as indicated in the Materials and Methods section. Optimal amount of rExoS-S100 proteins used: 2.4 µg rExoS129-S100A8, 3.75 µg rExoS129-S100A9, 3.75 µg rExoS129 alone, or a mix of 0.38 µg rS100A8 and 0.38 µg rS100A9. All recombinant ExoS-S100 fusion proteins were preincubated first with 500 nM CaCl_2_ for 20 min before adding first cytochrome *b_558_*, and then cytosol of EBV-B cells and then the other reagents. NADPH oxidase activity was expressed as turnover (s^−1^). Number of experiments n = 4 to 5. *p<0.05 versus control (NADPH oxidase activity of cytochrome *b_558_* plus 500 nM CaCl_2_ and EBV-B lymphocytes cytosol). (B) The effect of ExoS protein length fused to S100A8 or S100A9 was tested on NADPH oxidase activity in a similar cell free assay as described in Figure 4A but without addition of EBV-B lymphocytes cytosol. Recombinant ExoS129-S100A8, rExoS129-S100A9, rExoS54-S100A8, and rExoS54-S100A9 fusion proteins were expressed in *P. aeruginosa* and purified from the culture medium, after secretion. Recombinant rS100A8 and rS100A9 or rExoS30-S100A8 and rExoS17-S100A8 were expressed in *E. coli* after IPTG induction as previously described in Materials and Methods. Calcium (500 nM) loaded recombinant S100 proteins were used at the following optimal concentrations: ExoS129-S100A8 (2.4 µg), ExoS129-S100A9 (3.75 µg), ExoS54-S100A8 (5 µg), ExoS54-S100 A9 (5 µg), ExoS30-S100A8 (0.7 µg), ExoS17-S100A8 (0.8 µg) and rS100A8 or rS100A9 (0.38 µg each). NADPH oxidase activity was expressed as turnover (s^−1^). Number of experiments n = 6 *p<0.05 compared to control (NADPH oxidase activity of cytochrome *b_558_* plus 500 nM CaCl_2_). (C and D) The effect of different protein quantity of rExoS54-S100A8, rExoS54-S100A9, ExoS54-S100A8-A9 chimera, ExoS54-S100A9-A8 chimera, rS100A8 and rS100A9 was evaluated on NADPH oxidase activity after loading with 500 nM CaCl_2_, in a cell-free assay as described in Figure 4A and Figure 4B. Oxidase activity was expressed as turnover (s^−1^). Number of experiments n = 3 to 6. *p< 0.05 compared to control (NADPH oxidase activity of cytochrome *b_558_* plus calcium). Cyt *b_558_*, Cytochrome *b_558_*.

Recombinant rExoS-S100A8 and rExoS-S100A9 fusion proteins were collected from the cultured medium of EGTA induced bacteria and were tested *in vitro* on NADPH oxidase activity in a cell-free assay. We first evaluated the NADPH oxidase activity of the purified cytochrome *b*_558_ in the presence of the cytosol of EBV-B lymphocytes that contained p67^phox^, p47^phox^, p40^phox^, and Rac 1/2 as activating factors but no S100 proteins. Incubation was performed in the presence of 10 µM FAD, 40 µM GTPγS, and 5 mM MgCl_2._ The reaction was initiated by introducing 150 µM NADPH and an optimal amount of arachidonic acid (1 mM) as described in Materials and Methods. The addition of calcium loaded rExoS129-S100A8 or that of a (1/1) rS100A8 and rS100A9 mixture to cytochrome *b_558_* and then to cytosol and other reagents of the cell-free assay medium, led to a specific enhancement of NADPH oxidase activity up to 88±10 s^−1^ for rExoS129-S100A8 versus 59±3 for cytochrome *b_558_* plus cytosol, or 26±7s^−1^ for cytochrome *b_558_* alone (Figure 4A). Moreover, there was no effect of rExoS129-S100A9, of the 1/1 rExoS129-S100A8 and rExoS129-S100A9 or as expected without calcium which confirms the results obtained earlier with the EBV-B lymphocyte cells and could suggest that in the presence of rExoS129-S100A9, the conformation of rExoS129-S100A8 is lost or defective because of inadequate folding. Interestingly, as noticed in the Figure 3B in EBV-B lymphocyte cells, when fused with ExoS129, S100A8 becomes capable of stimulating the NADPH oxidase, as *in vivo* with S100A9 or *in vitro* with rS100A9 (Figure 4A).

We next investigated whether the stimulating effect of the S100 proteins could act directly on purified cytochrome *b_558_.* A similar experiment with recombinant fusion proteins and purified cytochrome *b*_558_ was performed but this time in the absence of EBV-B lymphocyte cytosol. Oxidase activity was measured as described in Figure 4A. The addition of calcium loaded rExoS129-S100A8 or rExoS54-S100A8 to cytochrome *b_558_* led to a specific enhancement of NADPH oxidase activity up to 58±7 s^−1^ and 43±6 s^−1^ respectively, versus control 26**±**5 s^−1^, contrary to what was observed with rS100A8 alone or with rExoS54-S100A9 (Figure 4B). The enhancement of oxidase turnover is dependent on rExoS54-S100A8 and rExoS54-S100A9-A8 chimera concentrations with an optimum at 2 µg (Figure 4C) and at 1.1 µg (Figure 4D) respectively. As expected, rExoS54-S100A9 (Figure 4C) and rExoS54-S100A8-A9 (Figure 4D) had no effect. Similar results were obtained with rExoS129-S100A8 and rExoS-129 S100A9 (not shown).

All these results highlight a specific interface between S100A8 and the cytochrome *b*_558_ and confirm that S100A9 is not involved in the intracellular activation of NADPH oxidase. They also suggest that the activation of NADPH oxidase is mediated by S100A8 *in vivo,* either by association with S100A9 in the presence of calcium, or *in vitro* by using a fusion protein with the first 54 or 129 N-terminal amino-acid residues of *P. aeruginosa* ExoS toxin; leading to the idea that the portion of ExoS fused with S100A8 may provide a proper conformation for S100A8 protein required for oxidase activation through cytochrome b558 interface. In order to determine the sequence of ExoS responsible for this effect, we therefore evaluated the NADPH oxidase activity in the presence of S100A9 or S100A8 fused with different length of ExoS (129, 54, 30 and 17 N-terminal amino-acid residues). Recently, the first 54 N terminal amino acids of ExoS was reported to correspond to the minimal domain required for a secretion by the *P. aeruginosa* [28], [30]. Therefore, since *P. aeruginosa* CHA strain is unable to secrete the S100 recombinant proteins fused with the ExoS30 or ExoS17, we produced those proteins (rExoS30- or 17-S100A8) in IPTG induced *E. coli,* as described in Material and Methods. A cell-free assay was performed by incubating purified cytochrome *b*_558_ with the various recombinant proteins expressed either from *P. aeruginosa* or *E. coli*. NADPH oxidase activity was measured after arachidonic acid stimulation. An optimum oxidase turnover of 58±7 s^−1^ was obtained with rExoS129-S100A8 versus control 28±5 s^−1^ as shown in Figure 4B; the stimulation effect disappeared when the length of the N-terminal domain of ExoS is shorter than 54 or is absent. Those results suggest that rExoS129 or rExoS54 may serve as a scaffold for the conformational required by S100A8 to stimulate the NADPH oxidase.

### The C-terminal Domain of S100A8 Proteins is Implicated in the NADPH Oxidase Activation

**Figure 5:**
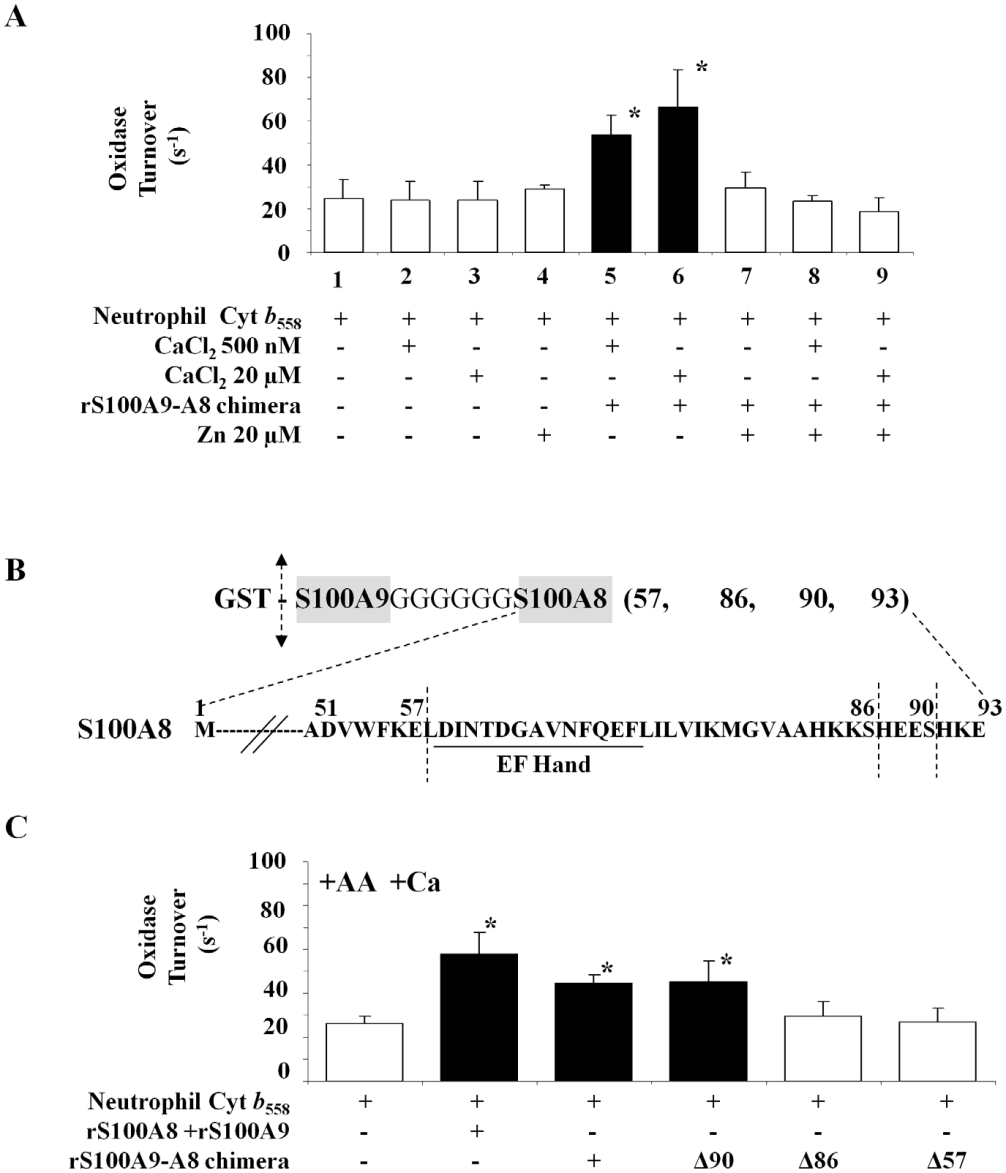
NADPH oxidase activation by rS100A9-A8 chimera full length and truncated forms. (A) Effect of zinc versus calcium on the NADPH oxidase activity measured in a cell-free assay as described in Figure 4 and in Materials and Methods. Cytochrome *b_558_* (0.2 pmol) was incubated in the presence (or not) of (20 µM or 500 nM) calcium loaded rS100A9-A8 chimera, and then with FAD, GTPγS, MgCl_2_ and an optimal amount of arachidonic acid (1 mM). The effect of pre-incubation of S100 chimera proteins with zinc versus calcium was tested on NADPH oxidase activity. The conditions are cytochrome *b_558_* incubate alone (1), with calcium (2–3), with zinc (4), with rS100A9-A8 chimera plus calcium (5–6), with rS100A9-A8 chimera plus zinc (7) and with rS100A9-A8 chimera plus calcium then plus zinc (8–9). NADPH oxidase activity was expressed as turnover (s^−1^). Number of experiments n = 6. *<0.05 compared to control (NADPH oxidase activity of cytochrome *b_558_* plus calcium). (B) Schematic representation of the C terminal sequence of full length and truncated S100A8 of the recombinant chimera proteins. The four chimera S100A9-full length S100A8 named rS100A9-A8, S100A9-truncated S100A8 named rS100A9-A8Δ90, S100A9-A8Δ86, or rS100A9-A8Δ57 were expressed in *E. coli* as described in Materials and Methods. (C) Recombinant rS100A9-A8, rS100A9-A8Δ90, rS100A9-A8Δ86, rS100A9-A8Δ57 chimeras were used in a cell-free assay as described in the Figure 4. The effect of each rS100 chimera proteins (1 µg) was compared to a mix of 1/1 rS100A9 and rS100A8 (0.38 µg), each S100 protein was preloaded with 500 nM CaCl_2_ for 20 min, before measuring NADPH oxidase activity and showed that the C-terminal domain of S100A8 is involved in the NADPH oxidase activation. NADPH oxidase activity was expressed as turnover (s^−1^). Number of experiments N = 5 to 8. *p< 0.05 compared to control (NADPH oxidase activity of cytochrome *b_558_* plus calcium). Cyt *b_558_*, Cytochrome *b_558_*.

We have demonstrated that S100A8 is a positive effector of NADPH oxidase (Nox2) activation (1) *in vivo*, after transfection of the genes encoding the S100A8 and S100A9 proteins [2], (2) *ex vivo* after injection of ExoS129 or rExoS54-S100A8 by *P. aeruginosa* to EBV B-lymphocytes, and (3) *in vitro* (cell-free assay) by using recombinant ExoS129 or rExoS54-S100A8 or a 1∶1 mixture of rS100A8 and rS100A9 proteins. We further wanted to analyze the role of different S100A8 domains on NADPH oxidase activity. Since S100A8 protein possesses EF-hand binding sites for the calcium and one motif of S100A8/S100A9 heterodimer zinc binding site, we first evaluated the implication of these two compounds in the S100A8-mediated NADPH oxidase activity. The effect of preloading with calcium versus zinc of S100 proteins was carried out to evaluate a putative role of zinc in mediating NADPH oxidase activation. First incubation with Zn^2+^ or Ca^2+^ alone (Figure 5A, lane 2, 3 and 4) did not modify the NADPH oxidase turnover. Recombinant rS100A9-A8 chimera protein, preloaded with 20 µM CaCl_2_ or only 500 nM, displays a significant enhanced oxidase activity, as shown on Figure 5A, lane 5 and 6. However, there was no activation when 20 µM ZnCl_2_ was added instead of calcium (Figure 5A, lane 7). The addition of first calcium and then zinc (Figure 5A, lane 8 and 9) or vice/versa (not shown), inhibits the oxidase activation by the rS100A9-A8 chimera protein. As reported previously [5], [20], it is likely that in the presence of both cations, Zn^2+^ replaced Ca^2+^ on the EF-hand binding sites and therefore annihilated the activation of NADPH oxidase by the rS100A9-A8 chimera protein.

In order to determine the domain of S100A8 involved in the stimulation of NADPH oxidase activity, several truncated forms of the rS100A9-A8 chimera proteins were designed to remove sequentially important domains of S100A8 necessary for their function. Therefore, the chimera rS100A9-A8 was truncated from the C terminal side to excise progressively the putative zinc motif and calcium binding domains of S100A8 (Figure 5B). Those proteins correspond to rS100A9-A8 full length or truncated at 57 (rS100A9-A8Δ57), 86 (rS100A9-A8Δ86), or 90 (rS100A9-A8Δ90) amino acid residues of S100A8 sequence. They were produced in *E. coli* (Figure 2) and purified proteins were identified by slot blot and Western blot as shown earlier (Figure 1D and E). These chimeras were incubated (1 µg each) after calcium loading (500 nM), with purified cytochrome *b*_558_ (0.2 pmol) in the presence of FAD, GTPγS, and MgCl_2_. NADPH oxidase activity, expressed as turnover (s^−1^), was measured after adding (150 µM) NADPH upon arachidonic acid stimulation as described in Materials and Methods. The results demonstrated that there was no effect of rS100A9-A8Δ86 or rS100A9-A8Δ57 contrary to rS100A9-A8 and rS100A9-A8Δ90 (Figure 5C). These data support the important role of the short sequence between the amino acid residues 86 and 90 of S100A8 protein for NADPH oxidase activation. This sequence was also reported to be part of zinc-binding site of S100A8/S100A9 heterocomplex [7].

### rS100A9-A8 Induces Active Conformational Change of Cytochrome *b_558_*

**Figure 6:**
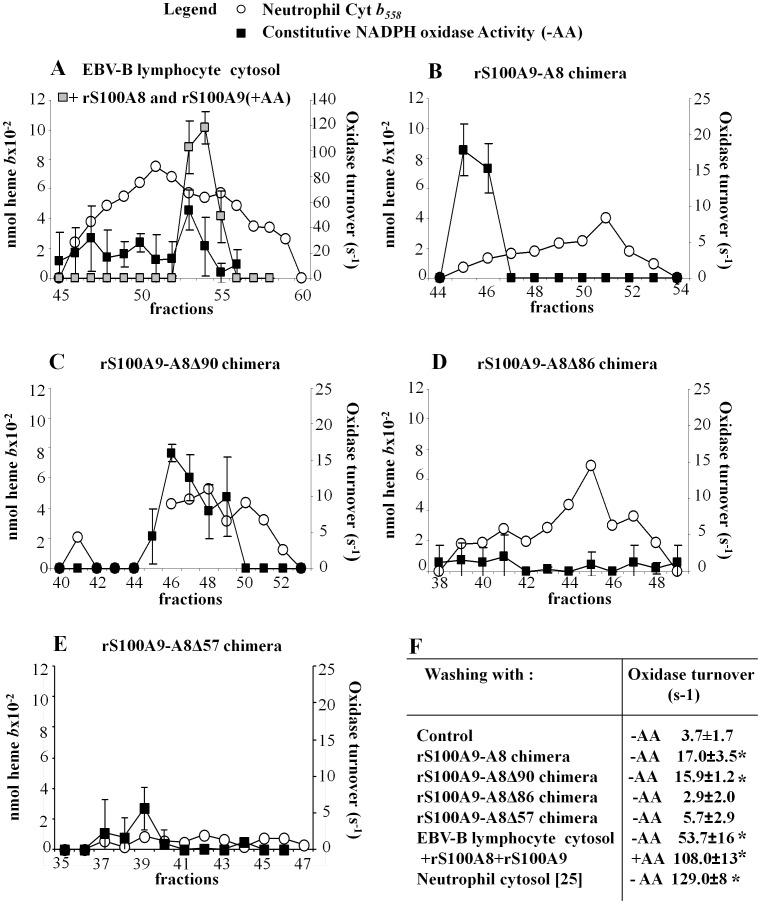
Constitutive NADPH oxidase turnover of the purified cytochrome *b_558_* isolated from stimulated neutrophils on heparin affinity matrix in the presence of rS100A9-A8 full length or truncated chimeras. Purification of cytochrome *b_558_* from stimulated neutrophils followed a standard protocol until it bound to the heparin affinity matrix as reported [25] and as described in Materials and Methods. The cytochrome *b_558_* bound to the affinity heparin matrix was washed with: (A) EBV-B lymphocyte cytosol; in some experiments, a 1/1 mixture of rS100A8 and rS100A9 was added to the Sephacryl eluted cytochrome *b_558_* and then the activity of NADPH oxidase was measured in the presence of (1 mM) arachidonic acid (grey square). (B) S100A9-A8; (C) S100A9-A8Δ90; (D) S100A9-A8Δ86; (E) S100A9-A8Δ57. S100 chimera proteins were preloaded with calcium (500 nM). After the washing step, eluted fractions containing cytochrome *b_558_* were pooled and filtrated on Sephacryl-S300. The concentration of cytochrome *b_558_* in the Sephacryl eluted fractions (open circle) was determined by measuring the “reduced minus oxidized” differential spectrum at 426 nm. NADPH oxidase activity of purified cytochrome *b_558_* was measured in a cell free assay with 0.2 pmol cytochrome *b*_558_/assay, in the presence of 10 µM FAD, 40 µM GTPγS, and 5 mM MgCl_2_ but without arachidonic acid, and after adding 150 µM NADPH. The NADPH oxidase activity was expressed as turnover (s^−1^) (black squares). No S100A9-A8 chimera or EBV-B lymphocyte cytosol was added to the heparin matrix, in control experiments. (F) Table representing the optimum NADPH oxidase turnovers obtained in A, B, C, D, E conditions, or by using cytosol of stimulated neutrophils instead of that of EBV-B lymphocytes [25]. Number of experiments n = 6 *p<0.05 compared to control (constitutive NADPH oxidase activity of purified cytochrome *b_558_* in control experiment).

Membrane cytochrome *b_558_* was extracted and then purified from stimulated neutrophils; it was isolated on a heparin affinity matrix (Figure S2). After washing the matrix with cytosol of either stimulated neutrophils or that of EBV-B lymphocytes, cytochrome *b_558_* was eluted and then filtrated on Sephacryl S-300. Purified cytochrome *b_558_* displayed a constitutive NADPH oxidase activity as previously described [25]. The optimum oxidase turnover (constitutive activity) was in the range of 129 s^−1^ with cytosol of neutrophils and 54 s^−1^ with cytosol of EBV-B lymphocytes versus 3.7 s^−1^ for the control (without washing). The oxidase turnover with the cytosol of EBV-B lymphocytes increased up to 108 s^−1^ after adding a 1/1 mixture of rS100A8 and rS100A9, and 1 mM arachidonic acid in the eluates (Figure 6A and Figure 6F). Cytochrome *b_558_* was also directly activated when the rS100A9-A8 chimera (pre-incubated with calcium) was used instead of cytosol, confirming that S100 proteins mediate the change of cytochrome *b_558_* conformation directly onto the heparin matrix. Cytochrome *b_558_* becomes active with an oxidase turnover of 17 s^−1^ versus 3.7 s^−1^ for the control (cytochrome *b_558_* without washing) Figure 6F. Similar experiments were carried out with rS100A9-A8 truncated chimera proteins as illustrated by Figure 6C, D, and E. The results revealed that the cytochrome *b_558_* constitutive oxidase activity depends on the length of S100A8. Only the rS100A9-A8Δ90 chimera activated cytochrome *b*_558_, whereas rS100A9-A8Δ86 and rS100A9-A8Δ57 chimeras did not. All together, these data strongly suggest that the C-terminal sequence of S100A8, between 86 and 90 amino acid residues, is necessary for NADPH oxidase activation through a specific interaction of S100 proteins with cytochrome *b_558_*.

### Direct Interaction of Cytochrome *b_558_* with Chimera S100A9-A8 Evidenced by Cross-linking Experiments

**Figure 7:**
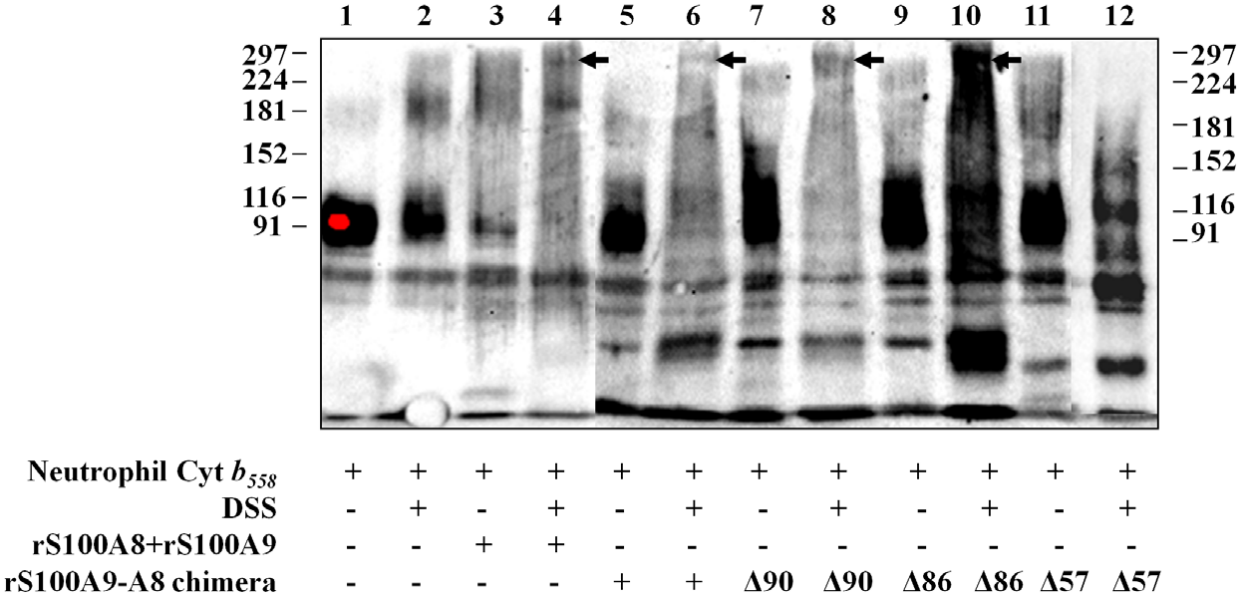
Disuccinimidyl suberate (DSS) complex formation between cytochrome *b_558_* and recombinant S100A8 and S100A9 proteins. Recombinant rS100A8 and rS100A9 proteins (1.5 nmol) or rS100A9-A8 full-length and truncated chimera proteins (1.5 nmol ) were pre-incubated with 500 nM calcium before adding 10 pmol of purified cytochrome *b_558_* in the presence of 10 µM FAD, 40 µM GTPγS, 5 mM MgCl_2_ and 1 mM of arachidonic acid and then 2 mM DSS (or not). After 1 h incubation at room temperature, the protein complexes were separated by 7% SDS-PAGE and electro transferred on nitrocellulose for Western blotting. Cytochrome *b_558_* was labeled with rabbit polyclonal antibodies raised against the C terminal sequence of gp91^phox^ [31]. The immune complexes were stained by ECL. The representative results represented correspond to one out of five experiments performed. Cyt *b_558_*, Cytochrome *b_558_*.

A dissuccinimidyl suberate, DSS cross-linking experiment was carried out with rS100A9-A8 chimera and cytochrome *b_558_* to investigate the molecular interaction between S100 proteins and cytochrome *b_558_*. A (1/1) mixture of rS100A8 and rS100A9, preloaded with calcium, was used in a preliminary experiment, to determine the optimum concentrations of DSS for cross-linking (not shown). A similar experiment was performed in the presence of cytochrome *b_558_*. Thus, purified and relipidated cytochrome *b_558_* (10 pmol) was incubated with 2 mM DSS. Calcium loaded S100 proteins were added to cytochrome *b_558_* in a medium containing FAD, GTPγS, MgCl_2,_ and then arachidonic acid, as described in Materials and Methods. Fractionation of proteins was performed by 7% SDS-PAGE followed by Western blot after incubation or not with DSS. The results illustrated by Figure 7 lanes 4 and 6, highlight a significant interaction of cytochrome *b_558_* with a 1/1 rS100A8 and rS100A9 mixture (revealed by the disappearance of the cytochrome *b_558_* band and the presence of complexed cytochrome *b_558_* and the S100 protein at the molecular size indicated by an arrow), or with rS100A9-A8 chimera versus control (lanes 3 and 5) or versus cytochrome *b_558_* without DSS (lane 1), or cytochrome *b_558_* plus DSS (lane 2). A similar experiment performed with rS100A9-A8Δ90 (lane 8) and with rS100A9-A8Δ86 (lane 10) showed an efficient cross-linking of the chimera with cytochrome *b_558_*, compared to that of rS100A9-A8Δ57 (lane 12), and to controls without DSS (Figure 7, lanes 7, 9 and 11). These data highlight the necessity of the C terminal domain of S100A8 in rS100A9-A8 chimera for the interaction with the cytochrome *b_558_* as previously suggested in the cell-free assay (Figure 5B).

### Co-localization of Endogenous S100A8/S100A9 and Cytochrome *b_558_* in PLB985

**Figure 8:**
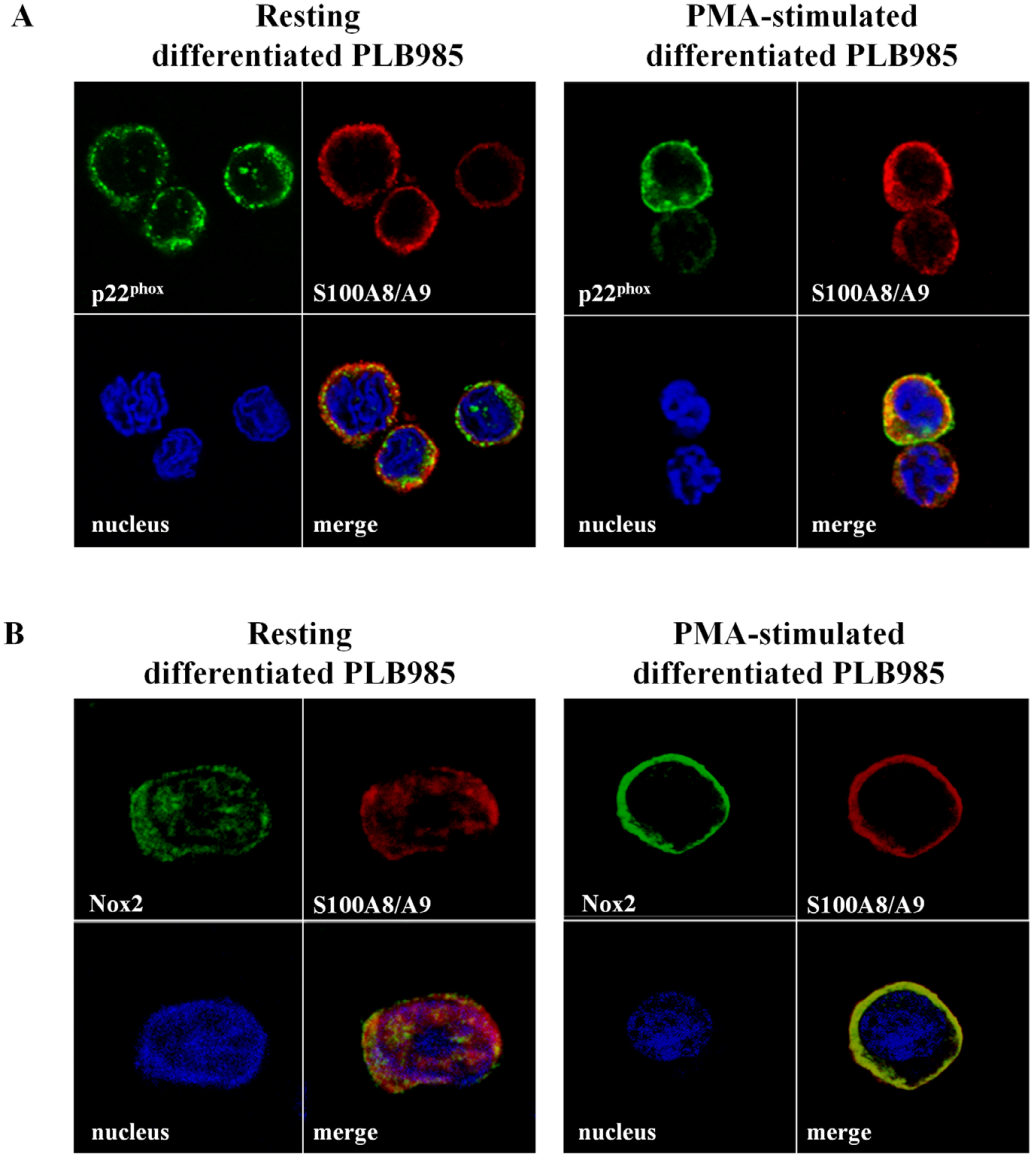
Co-localization of p22^phox^ or Nox2 with endogenous S100A8/S100A9 in PLB985 differenciated cells illustrated by confocal microscopy. Resting or PMA-stimulated differentiated PLB985 cells were incubated on poly-L-lysine-coated cover slips. They were fixed with 4% (w/v) paraformaldehyde and then permeabilized with 0.1% (w/v)×100 Triton as described in Materials and Methods. Permeabilized cells were co-labeled with a polyclonal anti-p22^phox^ IgG [31] and a 2H9 monoclonal antibody anti-S100A9 (A) or co-labeled with a 13B6 monoclonal anti-Nox2 and a polyclonal anti-S100A8/A9 [32] (B) to investigate the co-localization of cytochrome *b_558_* with endogenous S100A8/A9 proteins. A) Green, p22^phox^ and red, S100A8/A9. B) Green, Nox2 and red, S100A8/A9. Cell nuclei were stained in blue with Hoechst 33258. The figure represents one experiment out of three performed.

PLB985 cells were used to investigate, by confocal microscopy, the subcellular localization of endogenous S100A8/S100A9 proteins. Fixed differentiated PLB985 cells, unstimulated and PMA stimulated were permeabilised with 0.1% (w/v)×100 Triton and co-labeled with a rabbit polyclonal anti-p22^phox^ [31], or with a mouse monoclonal 13B6 anti-gp91^phox^ [32] and a mouse S100A8/S100A9 monoclonal antibodies (2H9). The results showed that endogenous S100A8/S100A9 heterocomplex in resting PLB985 (upper panel) was partitioned between cytosol and plasma membrane of PLB985 cells, while after PMA stimulation (lower panel), it translocated to the plasma membrane similar to p22^phox^ or gp91^phox^ revealed by a continuous labeling signal (yellow) along the plasma membrane which highlights the co-localization of the S100 proteins with p22^phox^ (Figure 8A) as with gp91^phox^ (Figure 8B).

## Discussion

We previously demonstrated, through *in vitro* and *ex vivo* approaches, that two proteins of the S100 family, S100A8 and S100A9, highly expressed in myeloid cells and mainly in neutrophils, are able to promote NADPH oxidase activation [2], [8], [25]. Our results strongly suggest a specific interaction of S100A8 with cytochrome *b*_558,_ the redox component of phagocyte NADPH oxidase, and introduce S100A8 as a privileged partner for NADPH oxidase activation. In this respect, a consensus site of the S100A8 C-terminal sequence is critical and should be directly involved in the interface with cytochrome *b_558_*.

*Pseudomonas aeruginosa* is an opportunistic bacterium; one of its major toxins, ExoS, is translocated into EBV-B lymphocytes through a type III secretion pathway [27]. This original procedure of injection into a deficient target cell allowed determining which of S100A8 or S100A9 is the main effector of NADPH oxidase activation. The *ex vivo* delivery of ExoS129-S100A8 fusion proteins activated NADPH oxidase of EBV-B lymphocytes as after transfection of the genes encoding for both S100A8 and S100A9 suggesting that S100A8 is the privileged partner of cytochrome *b*_558_. Interestingly, although it served primary as molecular “messenger” for protein delivery, ExoS also permitted a specific interaction of the injected protein with its partner suggesting that ExoS may facilitate a proper functional conformation for S100A8 [27]. However, this property disappears when the length of ExoS is shorter than 54 N terminal amino-acid residues. Rucks and Olson [33] reported that the N-terminal region of ExoS included a GTPase activating GAP domain [33]. The smallest GAP ExoS domain described is composed of the N-terminal 130 amino acid residues [34]. We confirmed that, *in vitro,* ExoS129-S100A8 and ExoS54-S100A8 had a similar enhancing effect on NADPH oxidase activity, while the helix involved in GAP activity was only present in the ExoS129 domain, and not in ExoS54. The data exclude any involvement of GAP activity in ExoS-S100A8 oxidase activation. The high affinity of S100A9 to S100A8 could stabilize S100A8 *in vivo* and facilitate the translocation to plasma membrane. The flexible ExoS of ExoS-S100A8 fusion protein could have a similar stabilizing conformation effect. Therefore, the 3D tertiary structure of the critical S100A8 domain involved in oxidase activation is probably similar in the calcium-loaded ExoS-S100A8 and in the calcium-loaded S100A8/S100A9 heterocomplex.

S100A8 and S100A9 are EF-hand molecules that need calcium to modulate target proteins. Cell calcium concentration is an important determinant for the biologic relevance of S100 proteins oligomerisation. The oligomeric status of S100A8 and S100A9 is rather complex in human: S100A8 and S100A9 are able to heterodimerize [21]. In the presence of calcium, they associate to form oligomers [35]. As a consequence, two different putative zinc-binding sites emerge at the S100A8/S100A9 subunit interface that may explain the well-known Zn^2+^ binding activity of hetero-complex. We observed that, not only S100A8/S100A9 heterocomplex (not shown) but also rS100A9-A8 protein chimera (this paper) do not activate NADPH oxidase after loading with zinc, as opposed to what is observed with calcium. These data confirm that only calcium is necessary for the S100A9/S100A8 heterocomplex to modulate the activity of the target cytochrome *b_558_*. Although, zinc and calcium bind to different consensus sites, our results suggest that in the presence of zinc, calcium is removed from its binding sites as previously reported [5], [20]. The transitory elevation of calcium in neutrophils after inflammatory stimuli, initiates reaction cascades beginning in cytosol with phosphorylation of the cytosolic activating factors of oxidase, mainly p47^phox^ and p67^phox^, and also with phosphorylation of S100A9 [2]. Phosphorylation could enhance S100A9 affinity for calcium as described recently for Nox5 [36], [37] and improve the Ca^2+^ loading of S100A8/S100A9 and translocation to plasma membrane [12]. However, the calcium source leading to its mobilization has not been formally identified.

S100A8/S100A9 is an intracellular reservoir for arachidonic acid in neutrophils [6]. Contrary to what was observed in the presence of calcium, arachidonic acid binding capacity to the S100 complex is not induced in the presence of zinc but may moreover reverse the effect of calcium [5]. However the concentration of Zn^2+^ used was within the range of 10 to 20 µM while, in cells and tissues, it is assumed to be below 1 µM; in consequence, this inhibition effect is negligeable and S100A8 and S100A9 may bind arachidonic acid *in vivo,* as both S100 proteins are calcium saturated [5]. When bound to arachidonic acid, S100A8/S100A9 changed tertiary structure conformation. Arachidonic acid is an anionic amphiphile which elicits oxidase activation in a cell-free system and which was reported to be a physiological activator of the NADPH oxidase in stimulated neutrophils [24]. A siRNA strategy supported the hypothesis that inducible phospholipase A2 controlled S100A8/S100A9 translocation and Nox2 activity, suggesting a close association between S100 proteins and the oxidase complex [12]. Furthermore**,** the plasma membrane concentration of arachidonic acid was locally enhanced upon assembly of NADPH oxidase complex. Thus, the long hydrophobic chain of arachidonic acid may facilitate the access of S100A8/S100A9 to cytochrome *b_558_* and favors activation of NADPH oxidase [38], [39].

S100A8/S100A9 complex potentiates activation of the phagocyte oxidase. It enhances the affinity of p67^phox^ to cytochrome *b_558_* in the presence of arachidonic acid and the change of cytochrome *b_558_* conformation, as highlighted by Atomic Force Microscopy, suggesting a specific molecular interface with the hemoprotein [2], [25]. The S100A8/S100A9 complex and p67^phox^ play a similar role in the oxidase activation process as being the signal that initiates, upon assembly of the oxidase complex, the change of both cytochrome *b_558_* conformation and electron transfer. We observed that co-localisation of S100 proteins and cytochrome *b_558_* differs at resting versus PMA stimulation and confirmed that assembly of the NADPH oxidase complex may proceed in microdomains as reported [40]. In fact, Jesaitis and coworkers reported that the change of cytochrome *b_558_* conformation may result from a 10Å movement of p22^phox^ away from the hemoprotein center. We have shown that the S100A8/S100A9 complex or the rS100A9-A8 chimera protein promotes activation of inducible (with arachidonic acid) or of constitutive (without arachidonic acid) NADPH oxidase. The data specifically highlight that S100A8 is directly involved in the activation process and that arachidonic acid is not enough to act by itself.

Finally, despite specificity of S100A8/cytochrome *b_558_*, interface, this interaction could be transitory with a rapid protein/protein dissociation process from an already active NADPH oxidase. Our cross-linking and co-localisation results suggest a molecular interaction between S100A8 protein and cytochrome *b_558_*. Four amino acid residues of the C-terminal sequence of S100A8, ^87^HEES^90^ are required and directly involved in this interface and are critical for oxidase activation. This portion has been shown to be involved in the dimerisation process that allows a correct conformation to the heterodimer S100A9/S100A8 [21]. Therefore, in its absence, the conformation of S100 protein could be disrupted and may explain the inefficiency of the truncated chimera rS100A9-A8Δ86 to stimulate NADPH oxidase activity.

These findings have implications for the mechanisms of NADPH oxidase activation. Arachidonic acid was proposed to mediate a transition between different coordinated forms of heme b that may induce the electron transfer to oxygen [10], [38]. A structural rearrangement of p22^phox^ mediated by anionic lipids was also suggested to increase the NADPH oxidase activity [40]. Kerkhoff’s group proposed a model in which S100A8/S100A9 was involved in the enhanced phagocyte NADPH oxidase activation by transferring the arachidonic acid to gp91^phox^ via an interaction with p67^phox^ [9]. We hypothesize that, additionally to the arachidonic acid effect on cytochrome *b_558_*, it is most likely that the S100A9-A8 chimera reported here or otherwise functional S100 oligomers interact specifically with the hemoprotein. The change of cytochrome *b_558_* conformation, mediated by this interaction, could expedite the electron transfer and enhance the affinity of p67^phox^ to cytochrome *b_558_*. Our data suggest further that the C-terminal sequence ^87^HEES^90^ of S100A8 plays an essential role in the interface, serving as a specific binding domain with a corresponding consensus site on cytochrome *b_558_*, the nature of which remains to be determined. However, the possibility that this sequence may be necessary for the formation of a correct and functional refolding of S100A8 itself, required to activate cytochrome *b_558_*, cannot be excluded.

## Materials and Methods

### Materials

Chemical reagents used in this study were obtained from the following sources: Heparin agarose, arachidonic acid, phorbol myristate acetate, carbenicillin, luminol (5-amino2, 3-dihydro-1, 4-phtalazinedione), monoclonal antibodies anti polyHistidine (Sigma Chemicals Co., St Louis, MO, USA); S100A8 (Calgranulin A, C-19) goat polyclonal antibody, donkey-anti-goat IgG antibody, S100A12 (Calgranulin C, 19F5) monoclonal antibody (Santa Cruz Biotechnology, Inc., Santa Cruz, CA, USA); Anti-goat and anti-rabbit IgG Alexa Fluor 488 or 546, or anti-mouse IgG Alexa Fluor 546 or 488 antibodies (Molecular Probes Europe BV Leiden, The Netherlands); PIA (DIFCO Laboratories, Detroit, MI, USA); ECL Western blot detection reagent, Sephacryl S-300 HR, diethylaminoethyl (DEAE) Sepharose CL-6B, CM-Sepharose CL-6B, N-amino octyl-Sepharose CL-4B,protein G Sepharose (Amersham Pharmacia Biotech, Uppsala, Sweden); N-octyl glucoside (Roche diagnostic, Meylan, France); DSS (Disuccinimidyl suberate) (Pierce Chemical Co, USA); Trizol® was from Invitrogen Life Technologies, Grand Island, NY , USA; Talon® Superflow^TM^ Metal Affinity was from Clonetech, Mountain View, Ca, USA; Nitrocellulose membrane 0.45 µm was from Bio-Rad Laboratories, Hercules, CA, USA. Polyclonal antibodies raised against S100 proteins purified from cytosol of neutrophils were described previously [2].

### Neutrophils, Lymphoid Cell Line, and PLB985 Cells

Buffy coat containing human neutrophils and B lymphocytes from healthy donor were purchased from the Etablissement Français du Sang (EFS, la Tronche, France) and were isolated according to previously described methods [41]. B lymphocytes were immortalized with the B95-8 strain of Epstein-Barr Virus (EBV) [42]; the EBV-B lymphocytes were cultured in RPMI 1640 supplemented with 10% (v/v) fetal calf serum, 2 mM L-glutamine at 37°C in 5% CO_2_ atmosphere. Cytosolic and membrane fractions from EBV-B lymphocytes and human neutrophils respectively, both cells being previously treated with 3 mM DFP, were prepared as described [31]. PLB985 cells [43] were grown in RPMI 1640 medium supplemented with 2 mM glutamic acid, 10% (v/v) fetal bovine serum, 100 U/ml penicillin, 100 µg streptomycin at 37°C and 5% CO_2_ atmosphere. PLB 985 cells (5×10^5^ cells/ml) were treated with 0.5% (v/v) DMF (1–6 days) for granulocyte differentiation [44].

### Bacterial Strains and Growth Conditions

The *Pseudomonas aeruginosa* wild type strain (CHA) was grown from a mucoid bronchopulmonary isolate, obtained from a cystic fibrosis patient (Grenoble University Hospital), in Luria-Bertani broth, at 37°C. After overnight culture, bacteria were suspended in Luria-Bertani medium; they were diluted to A_600 nm_ = 0.2 in the later medium containing 300 µg/ml carbenicillin, and then cultured for 1 to 5 h. *P*. *aeruginosa* type III secretion properties were induced *ex vivo* upon cell contact with serum opsonization (human AB serum) or *in vitro* upon calcium depletion by 5 mM EGTA in the presence of 20 mM MgCl_2_ [27].

### Plasmid Constructions (Table S1)

Plasmids, containing respectively the *ExoS-S100A8 and ExoS-S100A9 DNA* in frame fusion, were obtained by ligating the *ExoS XbaI-SalI* fragment and the *S100A8 SalI-SphI* or *S100A9 SalI-SphI* fragments in the *XbaI-SphI-*opened *E. coli-Pseudomonas* shuttle vector pUCP20, as previously described [27]. The ExoS clone that contained the coding sequence corresponding to the N-terminal amino acid of ExoS and Orf 1, its specific chaperone, was amplified by PCR, from the genomic DNA of the *P. aeruginosa* CHA strain [29]. *S100A8-SalI-SphI* and *S100A9-SalI-SphI* were obtained by reverse transcription PCR, using total RNA from fresh human neutrophils; they were confirmed by sequencing.

A *SalI-S100A8GGG-BamHI* and a *BamHI-GGGS100A9-SphI* fragment were developed by PCR and ligated in a *SalI-SphI* pUCP20 ExoS opened plasmid for the chimera fusion protein ExoS-S100A8-A9 plasmid construction. At the same time, the ExoS-S100A9-A8 plasmid was generated by ligation of *SalI-S100A9GGG-BamHI* and *BamHI-GGGS100A8-SphI* amplified fragments in the *SalI-SphI* pUCP20 ExoS plasmid.

A *S100A9-S100A8 SalI-NotI* fragment was developed by PCR, using the previous ExoS-S100A9-A8 plasmid. It was inserted in the *SalI-NotI* opened pGEX 5×2 *E. coli* protein production plasmid. Then, various forms of S100A9-A8 chimera proteins, truncated at amino acid 90, 86, and 57 of S100A8, were also generated and cloned in frame with GST in the pGEX 5×2 plasmid, instead of the full length S100A9-A8 chimera protein.

The ExoS17–S100A8 and ExoS30-S100A8 fusion proteins were also produced in *E. coli,* using the pGEX 5×2 plasmid. We extracted in *Xba-SalI* the *ExoS54 DNA* from the pUCP20 *ExoS54-S100A8 plasmid,* and replaced it by *ExoS17 Xba-SalI* or *ExoS30 Xba-SalI* fragment developed by PCR. Then *ExoS17-S100A8 or ExoS30-S100A8 BamHI-XhoI* fragments, obtained by PCR, were ligated in the *BamHI-XhoI* pGEX 5×2 linearized plasmid (Figure S2).

The cDNA encoding for S100A12 protein was obtained by Reverse Transcription of neutrophil total RNA extracted by TRIzol and amplified by PCR using specific primers including the cDNA encoding for the restriction site for *NdeI* and *XhoI* enzymes. The 305 pb cDNA obtained was ligated in the *NdeI-XhoI* opened pIVEX2.4d vector.

### Expression of Recombinant ExoS-S100A8 and ExoS-S100A9 Fusion Proteins by P. Aeruginosa (Figure 2 and Table S1)

Plasmids pUCP20 (empty vector), pUCP20-ExoS (expressing the 54 N-terminal or the 129 N-terminal residues of ExoS), pUCP20-ExoS-S100A8 and pUCP20-ExoS-S100A9, and fusion protein chimeras pUCP20-ExoS-S100A9-A8 or pUCP20-ExoS-S100A8-A9 were electroporated into CHA P. aeruginosa strains [27]. Secretion of recombinant proteins was induced by adding 5 mM EGTA when bacterial growth, evaluated by A_600 nm_, reached 0.6. Culture medium was supplemented with 20 mM MgCl_2_. After incubation at 37°C for 3 h, the induced culture suspensions were centrifuged at 12,000 g for 15 min at 4°C. The secreted proteins were pelleted down overnight with 55% (w/v) ammonium sulfate. After 12,000 g centrifugation (20 min, 4°C) the pellet was resuspended in 6 ml PBS and dialyzed overnight at 4°C. Proteins of the suspended pellet were fractionated by 11% SDS-PAGE. S100A8 and S100A9 proteins were immunodetected by Western blot using specific polyclonal antibodies raised against purified S100 proteins from cytosol of neutrophils [2].

### Delivery of S100A8 or S100A9 in EBV-B Lymphocytes by *P. Aeruginosa* Type Three Secretion System (TTSS)

The induced bacteria and EBV-B lymphocytes (8×10^7^ cells/ml) were suspended in RPMI 1640 medium containing 10% (v/v) human AB serum with a multiplicity of infection (MOI) of 10. Incubation at 37°C and mixing for 90 min allowed mutual adhesion. At the end of incubation, the mixture of bacteria and EBV-B lymphocytes was submitted to 300 g centrifugation. Bacteria remained in the supernatant. After washing with PBS, the pellet containing EBV-B lymphocytes was used to measure NADPH oxidase activity by chemiluminescence upon stimulation of 2×10^6^ cells with 130 nM PMA [27].

### Expression of Recombinant rExoS17-S100A8, rExoS30-S100A8, rS100A9 or rS100A8 or rS100A9-A8 Chimera Proteins, and rHis-S100A12 in *E.coli* (Figure 2 and Table S1)

BL21(DE3) competent E. coli were transformed with pGEX 5×2 containing DNA encoding for ExoS17-S100A8, ExoS30-S100A8, S100A9 or S100A8, and S100A9- full length or truncated A8 chimera proteins. Bacteria were grown in Luria-Bertani medium supplemented with 100 µg/ml ampicillin at 37°C, until A_600 nm_ reached 1.5. The production of recombinant proteins was induced by 0.2 mM IPTG, overnight, at 16°C. Bacteria were broken up by sonication and soluble GST fusion proteins were separated by ultracentrifugation at 100,000 g for 1 h and at 4°C. They were then affinity purified on glutathione-Sepharose in the presence of 10 mM DTT. After a washing step in PBS, recombinant proteins were cleaved directly on the matrix by using the XA factor in PBS overnight at 4°C. The matrix and soluble recombinant proteins were separated by filtration; recombinant proteins were stored at −20°C until further use.

pIVEX2.4d containing cDNA encoding for S100A12 in BL21(DE3) E. coli was used for the production of recombinant S100A12 in frame to 6 histidine residues at its N terminal side. Bacteria were lysed by sonication and the 10,000 g supernatant was ultra-centrifuged at 100,000g for 1 h and at 4°C. Soluble proteins were incubated for 1 h with Talon Metal affinity resins equilibrated in 50 mM Tris-HCl pH 7.5. The matrix was washed with 50 mM Tris-HCl pH 7.5 containing 100 mM imidazole, before elution of the bound rHis-S100A12 by previous buffer with 300 mM imidazole. The matrix and soluble recombinant proteins were separated by filtration; recombinant proteins were stored at −20°C until further use.

### Measurement of NADPH Oxidase Activity in EBV-B Lymphocytes by Chemiluminescence

Intact EBV-B lymphocytes (2×10^6^ cells), or EBV-B cells after injection of rExoS-S100A8, or rExoS-S100A9 or rExoS-S100 chimera fusion proteins, suspended in 50 µl PBS were added to 200 µl PBS containing 0.9 mM CaCl_2_, 0.5 mM MgCl_2_, 20 mM glucose, 20 µM luminol, and 10 U/ml horseradish peroxidase. Superoxide production was measured by chemiluminescence after adding 130 nM PMA [2]. Photon emission was recorded at 37°C for 1 h with a Luminoscan (Labsystem, Pontoise, France). Oxidase activity was expressed as relative light unit (RLU) per 2×10^6^ cells.

### NADPH Oxidase Activity Measurement in a Cell-free Assay with Purified Cytochrome *b*_558_

Cytochrome *b*_558_ was purified from the plasma membranes of 10^10^ PMA stimulated human neutrophils as previously reported [26]. It was then quantified by reduced-minus-oxidized difference spectra using an absorption coefficient of 106 mM^−1^.cm^−1^. Oxidase activity was reconstituted by incubating purified cytochrome *b*_558_ (0.2 pmol) that was previously relipidated with L-α-phosphatidylcholine II with or without cytosol (300 µg proteins) isolated from EBV-B lymphocytes in the presence of 10 µM FAD, 40 µM GTPγS, 5 mM MgCl_2,_ and an optimal amount of (1 mM) arachidonic acid in a final volume of 100 µl of PBS. In some experiments, a mixture of rS100A8 and rS100A9 or rExoS-S100 fusion proteins, preincubated for 20 min with 500 nM CaCl_2_ were added to cytochrome *b_558_* and then to cytosol, FAD, GTP(γ)S, MgCl_2,_ and arachidonic acid [2]. The reaction was initiated by introducing 150 µM NADPH in the medium. Oxidase activity was estimated by measuring the reduction of cytochrome c in the absence or the presence of superoxide dismutase at 550 nm (ε_550_ = 21.1 mM^−1^ cm^−1^), and was expressed as turnover (s^−1^) [45].

### Purification of Cytochrome *b*_558_ Activated by rS100A9-A8 (Full Length or Truncated) Chimera Proteins (Figure S1)

Purified membranes from PMA stimulated neutrophils were used for extraction of cytochrome *b*_558_ in the presence of 2% (w/v) n-octyl glucoside followed by fractionation of extracted proteins by various ion exchange chromatography columns, combined to heparin agarose affinity matrix as previously described [25]. The procedure of cytochrome *b_558_* purification was standard until it bound to the heparin affinity matrix. Briefly, the protein fractionation from soluble extract was carried out in the presence of 0.1% (w/v)×100 Triton first onto anion exchange columns prepared with a mixture (1v/1v/1v) of CM-Sepharose, DEAE Sepharose, and N-amino-octyl-Sepharose, and then onto a heparin agarose affinity matrix. Cytochrome *b*_558_ bound to the later matrix. At this stage,×100 Triton was replaced by 40 mM n-octyl glucoside added to the purification buffer. Cytosol of stimulated or non stimulated EBV-B lymphocytes (10 mg proteins), or intact or truncated rS100A9-A8 (3 mg) chimera preloaded with 500 nM CaCl_2_ for 20 min, were applied to the matrix for extensive washing [8], [25]. Cytochrome *b*_558_ bounded to the heparin agarose was eluted using a NaCl gradient ranging from 0 to 0.5 M. The presence of cytochrome *b*_558_ was detected in the elution fractions by measuring the “reduced minus oxidized” differential spectrum. It was quantified at 426 nm using a ε_426_ value of 106 mM^−1^ cm^−1^. The eluates containing cytochrome *b_558_* were pooled and filtrated on Sephacryl S-300 column. NADPH oxidase activity of the final fraction was measured in a cell-free assay with 0.2 pmol of the later purified cytochrome *b_558_*/assay in the presence of 10 µM FAD, 40 µM GTPγS, and 5 mM MgCl_2_. The reaction was initiated by adding 150 µM NADPH, but without any stimulation by arachidonic acid; activity was expressed as turnover (s^−1^) [25]. A control experiment was performed without any washing of the heparin matrix.

### Covalent Cross-linking

The method was described previously [46]. Briefly, 10 pmol of purified and relipidated cytochrome *b_558_* were incubated with 1.5 nmol of S100 proteins: the four rS100A9-A8 chimeras, or a 1/1 mixture of rS100A8 and rS100A9. rS100 proteins were preincubated in a 500 nM CaCl_2_ medium for 20 min before adding: first 10 pmol of cytochrome *b_558_*, then 10 µM FAD, 40 µM GTPγS, 5 mM MgCl_2_, and finally 1 mM arachidonic acid. After 10 min of incubation at room temperature, 2 mM of Disuccinimidyl Suberate (DSS) was added (or not). After 1 h of incubation at room temperature, the cross linking was stopped by adding 10 mM Tris-HCl pH 7.5 for 10 min. Protein samples were incubated in a denaturating sample buffer [47] containing 2% SDS and 2.5% β-mercapto-ethanol. Fractionation of complexed proteins was performed by SDS-PAGE (7%), followed by Western blotting using rabbit polyclonal antibodies raised against the C-terminal peptide of gp91^phox^ [31].

### Confocal Microscopy

Confocal microscopy was carried out as previously described [32], [44]. Briefly, control EBV-B lymphocytes, *P. aeruginosa* incubated-EBV-B lymphocytes, and 6 days differentiated PMA-stimulated or unstimulated PLB985 cells (2×10^5^ cells in PBS) were coated onto a poly-L-lysine- round glass cover-slip, before fixing with 4% (w/v) paraformaldehyde during 10 min for EBV-B lymphocytes or during 20 min for PLB985 cells. The cells were permeabilised with 0.1% (w/v)×100 Triton (1 min at room temperature). After an extensive washing with PBS, and quenching the fluorescence of paraformaldehyde by 50 mM NH_4_Cl, the cells were subsequently incubated during 1 h for EBV-B lymphocytes or during 45 min for PLB985 cells with primary antibodies as follows: polyclonal goat anti-S100A8 antibodies (2 µg), purified monoclonal anti-S100 antibodies 2H9 (1,9 µg), rabbit polyclonal anti-p22^phox^ [31], mouse monoclonal anti-Nox2 13B6 [32], rabbit polyclonal anti-S100A8/A9 [25]; followed by 45 min incubation with secondary antibodies anti-goat Alexa Fluor 488, anti-mouse Alexa Fluor 546, Alexa Fluor 488 or anti-rabbit Alexa Fluor 488 or Alexa Fluor 546 (1∶1,000). Cell nuclei were stained with Hoechst 33258. Fixed cells were imaged at room temperature using the inverted confocal and two-photon laser-scanning microscope (LSM 510 NLO, Carl Zeiss) equipped with a 40×/1. 3 Plan-Neofluar oil immersion lens [44].

### Flow Cytometry

Undifferenciated or 6 days differentiated PLB985 cells (10^7^ cells/ml in PBS) were fixed by paraformaldehyde 2% (v/v) for 15 min at 4°C. The cells were washed and resuspended in PBS containing 0.5 mM CaCl_2_ and 0.2% (w/v) BSA. They were permeabilised with 0.01% (w/v) saponin for 10 min on ice. Monoclonal antibodies raised against S100 proteins of neutrophil cytosol, 5A10 or 2H9 (5 µg each) were added to 10^6^ cells that were differentiated or not for 30 min at 4°C. Irrelevant IgG2a and IgG1 (5 µg each) were used as controls, for 2H9 and 5A10 respectively. After washing, anti-mouse secondary antibodies conjugated to phycoerythrin were used for the fluorescence detection. Fluorescence was measured on a FACScalibur cytomoter (Becton Dickinson).

### Production of Monoclonal Antibodies Against S100A8 and S100A9

Mice immunization, monoclonal antibody production, and isotype identification were performed by Hybrisere (Grenoble, France). Briefly 4 mice were immunized by 4 peritoneal injections of S100 proteins (10 µg) purified from neutrophils cytosol [2] and pre-incubated with 500 nM CaCl_2_. The mice sera were tested by ELISA. Hybridization was performed after a supplementary injection. Monoclonal antibodies from hybridoma were evaluated by ELISA and produced in ascetic fluid. Two clones were selected: 5A10 (IgG1) and 2H9 (IgG2a).

IgGs were produced from ascetic fluid. After centrifugation at 10,000 g for 10 min, at 4°C, IgGs were precipitated from supernatant by 50% (w/v) saturation ammonium sulfate for 10 min. The pellet was resuspended in 100 mM borate buffer pH 8.9 containing 3 M NaCl for IgG1, or in 50 mM borate buffer, pH 8.9, 3 M NaCl for IgG2a and dialyzed overnight. IgG2a was purified on Protein G Sepharose equilibrated in the same buffer, and after an extensive washing of the matrix, it was eluted with 0.1 M Glycine pH 3; then the pH was readjusted with 1 M Tris to pH 8.5. IgG1 bound to Protein G Sepharose in 100 mM borate, 3 M NaCl pH8.9, and was washed first with 50 mM borate buffer, 3 M NaCl pH 8.9 and second with 10 mM borate buffer, 3 M NaCl pH 8.9 before the elution in 0.1 M Glycin pH 3. The pH was then equilibrated to pH 8.5 with 1 M Tris. The presence of IgG in the fractions was measured at 280 nm and purity was controlled by 11% SDS PAGE.

### Slot Blot

Purified rS100A8, rS100A9, or rS100A12 proteins, or purified S100 proteins from cytosol of neutrophils (1.25 µg or 5 µg), or rS100A9-A8 full-length or truncated chimeras (5 µg) were adsorbed on nitrocellulose membrane under vacuum. The experiment was carried out in the Bio Dot system (Bio-Rad) as previously described [32]. After adding 1% (w/v) low-fat milk proteins, in TBS/0.05% Tween for membrane saturation, nitrocellulose was incubated with the primary antibodies, non-immune, 2H9, 5A10, 19F5, or polyclonal antibodies raised against S100 proteins from cytosol of neutrophils (1∶1,000 each) and then with secondary antibody labeled with peroxidase (1∶5,000). Peroxidase activity was detected by ECL.

### SDS-PAGE and Western Blot

Proteins were fractionated by 7%, 11%, 12.5%, or 15% SDS-PAGE [47] and electrotransferred to nitrocellulose [48]. Immunodetection was performed using rabbit polyclonal antibodies raised against S100 proteins purified from cytosol of neutrophils (IgG dilution, 1∶1,000), or anti peptide polyclonal antibodies raised against gp91^phox^, (serum dilution, 1∶500). When necessary, monoclonal antibodies were used as follows: 2H9, 5A10, 19F5, monoclonal anti-histidine, 1∶1,000 each. Immune complexes were detected with goat secondary antibody combined with peroxidase (1∶5,000). Peroxidase was detected by ECL.

### Toxicity of the CHA Strain Assessment

Toxicity of the CHA strain was assessed by measuring lactate dehydrogenase activity in the medium: it started after 2.5 h of contact with normal EBV-B lymphocytes [49].

### Statistical Analysis

Data represent means±SD. ANOVA and a test a posteriori PLSD Fisher was used to determine the statistical significance of the results (p<0.05).

## Supporting Information

Figure S1 **Schematic representation of the isolation of active cytochrome** ***b_558_*** **from heparin agarose matrix procedure.** Cytochrome *b_558_* was extracted with 2% (w/v) octyl-glucoside from the membrane of stimulated human neutrophils as described in Materials and Methods. Proteins of the soluble extract were loaded onto a mixture of CM, DEAE, and n-amino-octyl Sepharose combined to heparin agarose. Cytochrome *b_558_* bound to the heparin affinity matrix. The matrix was extensively washed with either rS100A9-A8 chimera or with cytosol of stimulated EBV-B lymphocytes. The cytochrome *b_558_* containing fractions eluted from heparin agarose were pooled and filtrated on S-300 Sephacryl. Purified cytochrome *b_558_* recovered from Sephacryl displayed a constitutive NADPH oxidase activity.
(TIF)

Figure S2 **Purification and identification of rHis-S100A12.** (A) Recombinant rHis–S100A12 was affinity purified from the 100,000 g supernatant of IPTG induced BL21 (DE3) *E. Coli* lysis medium as described in Materials and Methods. U stands for the 100,000 g supernatant; E1 to E4 are the 300 mM imidazole eluted fractions from the Talon matrix. Proteins of samples U and E were fractionated by 15% SDS-PAGE and stained with Coomassie Blue. (B) rHis-S100A12 proteins were identified by Coomassie bleu staining and by Western blot with a monoclonal antibody anti-S100A12 (19F5) or a monoclonal anti-histidine antibody. Cyt *b_558_*, Cytochrome *b_558_*.
(TIF)

Table S1 **Plasmid construction for protein expression.** cDNA encoding for S100A8, S100A9, S100A12 or fusion chimera proteins were introduced in pUCP20, pGEX5x2 or pIVEX2.4d plasmids using the indicated restriction enzyme. Protein expression was carried out in *Pseudomonas aerugina* (1) or *Escherichia coli* (2).
(DOC)

Table S2 **Evaluation of EBV-B lymphocytes viability incubated with** ***Pseudomonas aeruginosa*** **by Lactate Dehydrogenase (LDH) activity.** LDH activity was evaluated in the incubation medium after 90 min incubation of EBV-B lymphocytes with Pseudomonas aeruginosa or not. Unit represents the number of µmol of substrate transformed per min in 200 µl of contact medium.
(DOC)
