# Supplementary figures and images for: Molecular Interface of S100A8 with Cytochrome b558 and NADPH Oxidase Activation
Source: PLoS One. 2012 Jul 10;7(7):e40277. doi: 10.1371/journal.pone.0040277 (PMC3393751; doi:10.1371/journal.pone.0040277)

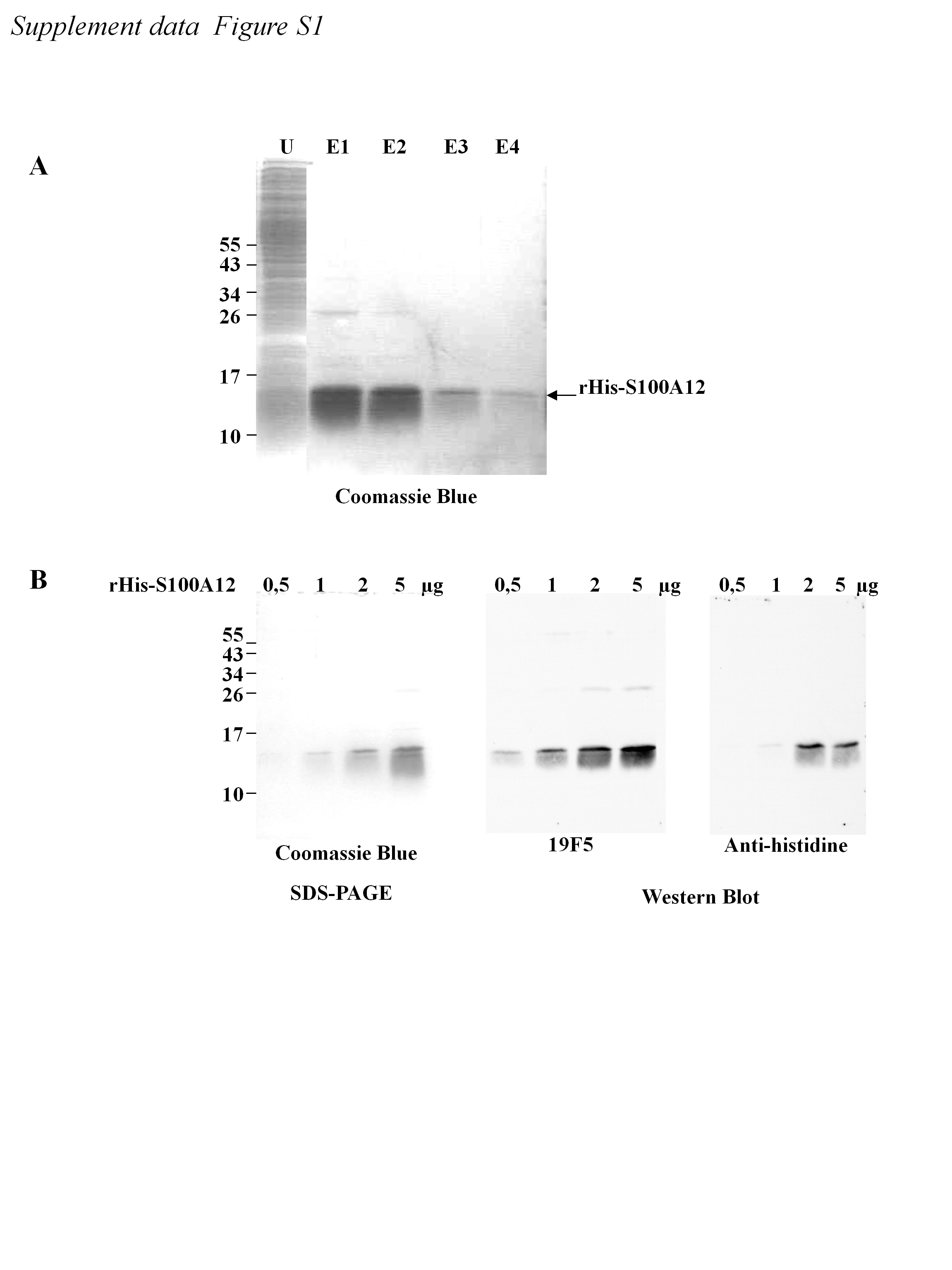

Supplement: Figure S1 — Schematic representation of the isolation of active cytochrome b558 from heparin agarose matrix procedure. Cytochrome b558 was extracted with 2% (w/v) octyl-glucoside from the membrane of stimulated human neutrophils as described in Materials and Methods. Proteins of the soluble extract were loaded onto a mixture of CM, DEAE, and n-amino-octyl Sepharose combined to heparin agarose. Cytochrome b558 bound to the heparin affinity matrix. The matrix was extensively washed with either rS100A9-A8 chimera or with cytosol of stimulated EBV-B lymphocytes. The cytochrome b558 containing fractions eluted from heparin agarose were pooled and filtrated on S-300 Sephacryl. Purified cytochrome b558 recovered from Sephacryl displayed a constitutive NADPH oxidase activity. (TIF) [file pone.0040277.s001.tif]

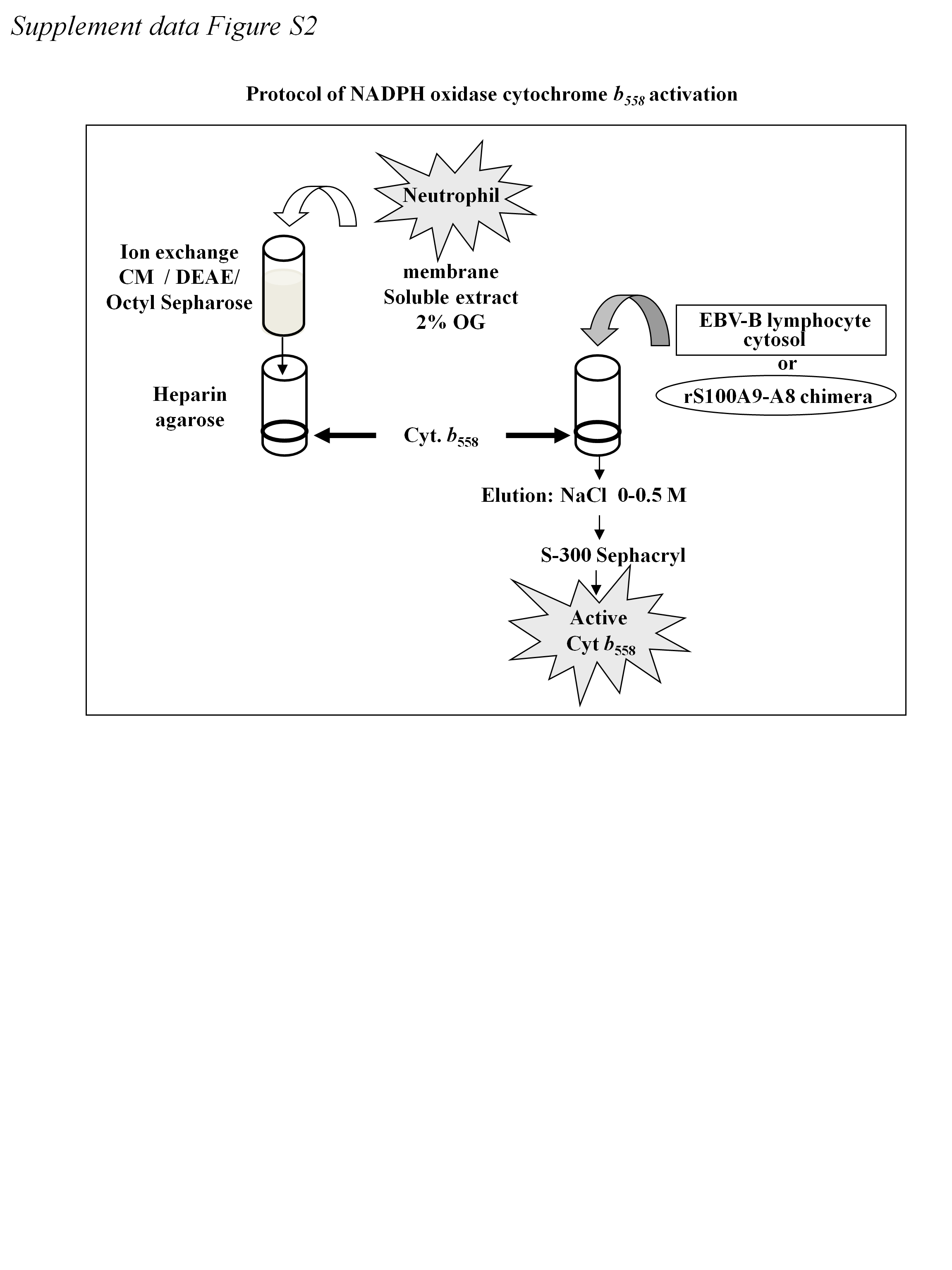

Supplement: Figure S2 — Purification and identification of rHis-S100A12. (A) Recombinant rHis–S100A12 was affinity purified from the 100,000 g supernatant of IPTG induced BL21 (DE3) E. Coli lysis medium as described in Materials and Methods. U stands for the 100,000 g supernatant; E1 to E4 are the 300 mM imidazole eluted fractions from the Talon matrix. Proteins of samples U and E were fractionated by 15% SDS-PAGE and stained with Coomassie Blue. (B) rHis-S100A12 proteins were identified by Coomassie bleu staining and by Western blot with a monoclonal antibody anti-S100A12 (19F5) or a monoclonal anti-histidine antibody. Cyt b558, Cytochrome b558. (TIF) [file pone.0040277.s002.tif]
