# Supplementary material for: Molecular Interface of S100A8 with Cytochrome b558 and NADPH Oxidase Activation
Source: PLoS One. 2012 Jul 10;7(7):e40277. doi: 10.1371/journal.pone.0040277 (PMC3393751; doi:10.1371/journal.pone.0040277)
Supplement: Table S1 — Plasmid construction for protein expression. cDNA encoding for S100A8, S100A9, S100A12 or fusion chimera proteins were introduced in pUCP20, pGEX5x2 or pIVEX2.4d plasmids using the indicated restriction enzyme. Protein expression was carried out in Pseudomonas aerugina (1) or Escherichia coli (2). (DOC) [file pone.0040277.s003.doc]

**Plasmids sequence for protein Insertion of fragments and restriction sites**

**and restriction enzymes**

1- In ***Pseudomonas aeruginosa***

pUCP20 *XbaI* *orf I – exoS 129* *SalI* *SalI*  *S100A8* *SphI*

*SalI* *S100A9* *SphI*

*SalI* *S100A9GGG* *BamHI* GGGS100A8 *SphI*

*SalI* *S100A8GGG* *BamHI* GGGS100A9 *SphI*

*XbaI* *orf I – exoS 54* *SalI* *SalI S100A8 SphI*

*SalI S100A9 SphI*

*SalI S100A9GGG BamHI GGGS100A8 SphI*

*SalI S100A8GGG BamHI GGGS100A9 SphI*

2- In ***Escherichia coli***

pGEX 5x2 GST *XbaI exoS 30 SalI S100A8 XhoI*

*XbaI exoS 17 SalI S100A8 XhoI*

pGEX 5x2 GST  *SalI S100A9GGG BamHI GGGFL S100A8NotI*

***S****alI S100A9GGG BamHI GGGΔ90 S100A8 NotI*

*SalI S100A9GGG BamHI GGGΔ86 S100A8 NotI*

*SalI S100A9GGG BamHI GGGΔ86 S100A8* *NotI*

pGEX 5x2 GST  *SalI S100A8 NotI*

*SalI S100A9 NotI*

pIVEX2.4d 6His *NdeI S100A12 XhoI*
