# Supplementary material for: Molecular Interface of S100A8 with Cytochrome b558 and NADPH Oxidase Activation
Source: PLoS One. 2012 Jul 10;7(7):e40277. doi: 10.1371/journal.pone.0040277 (PMC3393751; doi:10.1371/journal.pone.0040277)
Supplement: Table S2 — Evaluation of EBV-B lymphocytes viability incubated with Pseudomonas aeruginosa by Lactate Dehydrogenase (LDH) activity. LDH activity was evaluated in the incubation medium after 90 min incubation of EBV-B lymphocytes with Pseudomonas aeruginosa or not. Unit represents the number of µmol of substrate transformed per min in 200 µl of contact medium. (DOC) [file pone.0040277.s004.doc]

**EBV-BL incubated with** **LDH**

**U/min**

Alone 0,162

CHA 0,177

CHA pUCP20 0,160

CHA pUCP20-ExoS129-S100A8 0,165

CHA pUCP20-ExoS129-S100A9 0,154

CHA pUCP20-ExoS129-S100A8 + 0,156

CHA pUCP20-ExoS129-S100A9

CHA pUCP20-ExoS129-S100A9-A8 chimera 0,150

**Bacteria LDH**

**U/min**

CHA 0,005

CHA pUCP20 0,005

CHA pUCP20-ExoS129-S100A8 0,005

CHA pUCP20-ExoS129-S100A9 0,005

CHA pUCP20-ExoS129-S100A8-A9 chimera 0,005
